# Direct Growth of BODIPY‐Based Conjugated Porous Polymer Shell on Upconversion Nanoparticles for NIR‐Driven Photocatalysis

**DOI:** 10.1002/smll.75481

**Published:** 2026-09-10

**Authors:** Teresa Naranjo, Laura Francés‐Soriano, Delia Bellezza, Julia Pérez‐Prieto, Víctor A. de la Peña O´Shea, Marta Liras, María González‐Béjar

**Affiliations:** ^1^ Photoactivated Processes Unit IMDEA Energía Madrid Spain; ^2^ Instituto De Ciencia Molecular (ICMol)/Departamento De Química Orgánica Universitat De València Valencia Spain

**Keywords:** conjugated porous polymers (CPPs), nanohybrid, photochemistry, photophysics, upconversion

## Abstract

The great applications of upconversion nanoparticles (UCNPs) can be further extended by their encapsulation within conjugated porous polymers (CPPs). However, the synthesis of monodisperse core@shell based CPPs upconversion nanohybrids remains a challenge. Herein, we report a simple, general strategy that combines (i) prefunctionalization of UCNPs with polymerizable units, (ii) monomer‐feed control, and (iii) a centrifugation‐based work‐up to ensure anchoring to the inorganic core. This approach enables covalent growth of CPP shells with tunable thickness and low polydispersity. Thus, a BODIPY‐based CPP shell was grown over both core (NaYF_4_: Yb^3+^ (20%), Er^3+^ (2%)) and core‐shell (NaYF_4_: Yb^3+^ (20%), Er^3+^ (2%)@NaYF_4_) UCNPs, yielding UC_C_@CPP and UC_CS_@CPP nanohybrids. Under near‐infrared (NIR) excitation, the UCNP core upconverts light to activate the surrounding CPP shell leading to photocatalytic activity. UC_CS_@CPP exhibits a twofold higher photocatalytic activity than UC_C_@CPP highlighting the critical role of the NaYF_4_ passivation layer in preserving upconversion efficiency. Our approach overcomes key limitations of previous designs achieving improved photooxidation yields (i.e., 42%) and excellent recyclability. This modular platform provides a robust and versatile route to UC@CPP hybrids for designing NIR‐driven, reactive oxygen species (ROS)‐generating photocatalysts.

## Graphical abstract

Monomer prefunctionalization of upconversion nanoparticles directs uniform growth of BODIPY‐based conjugated porous polymers shells, affording precise control over polymer loading and optimized NIR‐triggered photocatalytic activity.

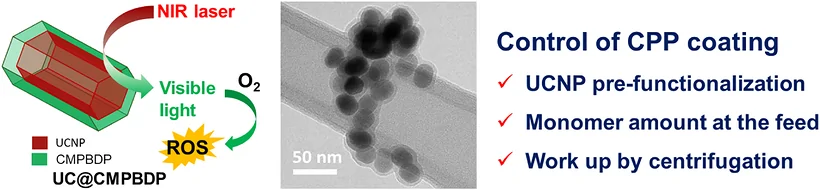

## 1 Introduction

Upconverting nanoparticles (UCNPs) have emerged as tunable photoactive materials owing to their ability to convert lower‐energy near‐infrared (NIR) light into higher‐energy NIR, visible or ultraviolet light via multiphotonic and non‐linear processes [1]. Their unique photophysical properties, including narrow emission bands, long luminescence lifetimes, high photostability, and low cytotoxicity, have enabled applications across various fields, such as bioimaging, theranostics, solar cells, and photocatalysis [2]. Furthermore, UCNPs provide a robust platform to build multifunctional upconversion nanohybrids (UCNHs) through surface functionalization with complementary photoactive components [3]. These UCNHs are expected to show enhanced properties arising from the synergy of both materials [3, 4].

In photocatalysis, the optimization of the synthetic protocol used to prepare UCNHs (e.g., number and aggregation state of photoactive molecules anchored to the UCNP) is crucial for achieving the best photocatalytic performance due to their influence on the photophysical events occurring upon NIR irradiation [5, 6]. However, some of these nanohybrids suffer from leaching [5] and new synthetic methods are being developed to achieve stronger/more stable interactions between the UCNP surface and its photoactive capping, e.g., in situ cation exchange to build nanoheterostructures [7].

In this context, porous organic polymers (POPs) in both versions, conjugated (micro)porous polymers (CMPs/CPPs) [8, 9] as porous amorphous materials and Covalent Organic Frameworks (COFs) as porous crystalline version, are of extreme relevance due to their extended π‐conjugated networks, hypercrosslinked molecular structure, intrinsic porosity, and inherent robustness under irradiation. Furthermore, their electronic structure can be easily tuned at the molecular level by modulating the nature of the organic building blocks [10].

Several approaches have been reported to combine POPs with inorganic nanoparticles of different nature [11, 12]. However, POPs have recently been integrated with UCNPs to develop photoactive nanohybrids that combine the tunable porosity and conjugation of POPs with the unique optical properties of UCNPs. Although still limited in number, the reported synthetic approaches can be classified into three main strategies (Figure 1). The first one consists of embedding UCNPs within a continuous POP network to form nanocomposites (Figure 1A) [13, 14, 15]. The second one relies on the solvothermal growth of COFs around surface‐modified UCNPs with a SiO_2_ layer, leading to submicrometric UCNP‐COF composites (Figure 1B) [16]. Finally, well‐defined UCNP@COF core@shell structures have been obtained by coating UCNPs with intermediate polymeric layers that direct the crystallization of the COF shell (Figure 1C).

**FIGURE 1:**
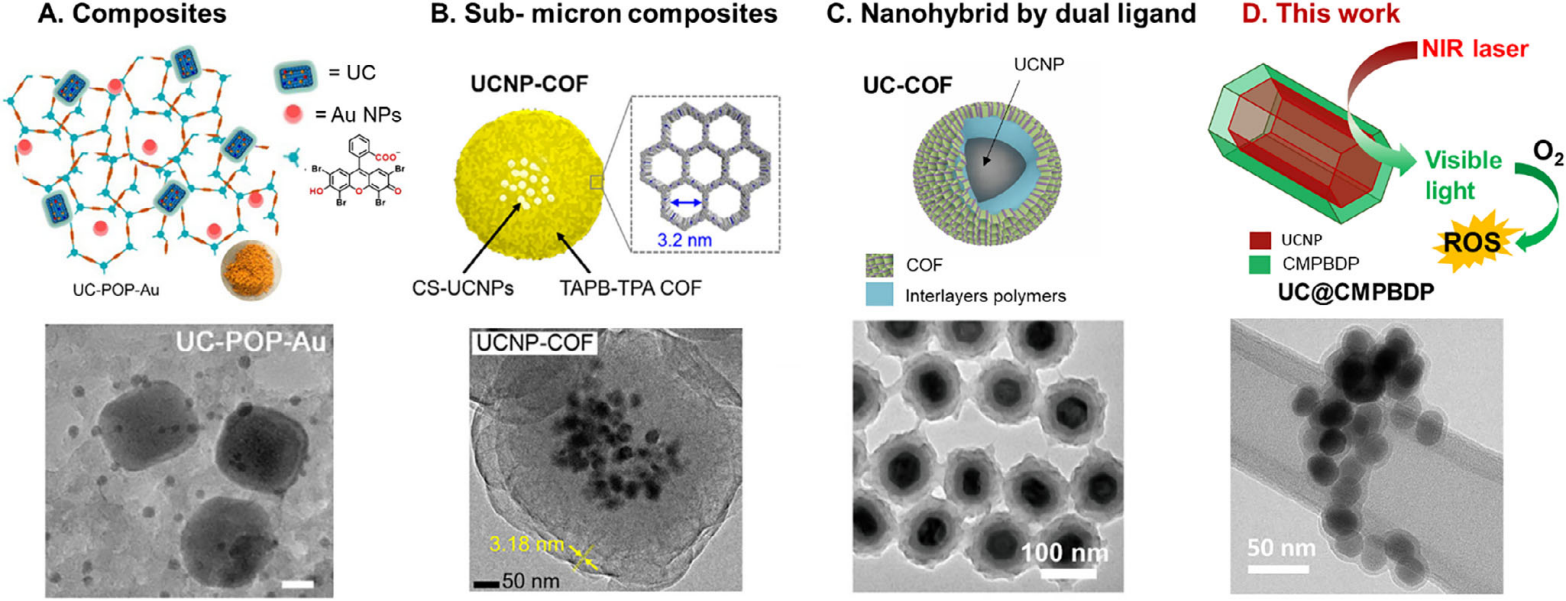
Comparative synthetic strategies from literature with this work. (A) Composites. Adapted with permission from ref. [15] copyright 2023 Wiley and Sons; (B) Sub‐micron composites. Adapted with permission from ref. [16] copyright 2025, Wiley and Sons; (C) Core@shell nanohybrid synthesized by dual‐ligand assistant encapsulation strategy. Adapted with permission from ref. [20] copyright 2021, Nature publishing group; (D) This work, based on the prefunctionalization strategy. Schematic illustration of NIR laser‐activated Reactive Oxygen Species (ROS) production by UC@CMPBDP core@shell nanohybrid (Top) and TEM photograph showing morphology (Bottom).

Despite these advances, the reported strategies still present important limitations. Nanocomposite architectures frequently suffer from heterogeneous nanoparticle distribution and partial pore blocking, which can reduce the effective interaction between the UCNPs and the conjugated polymer network [17, 18]. Conversely, core@shell COF systems generally require intermediate non‐conjugated coatings (e.g., polyacrylic acid (PAA), polyethyleneimine (PEI), or polyvinylpyrrolidone (PVP), preventing direct contact between the UCNP surface and the photoactive porous framework and often resulting in shells with non‐uniform thickness [19, 20].

To overcome these limitations, we adopted a surface‐prefunctionalization strategy based on naked UCNPs, inspired by a previous report on non‐porous polymer coatings [21]. In our approach, 4‐bromobenzenesulfonic acid (BBS) acts simultaneously as anchoring ligand and polymerization handle, enabling the direct Sonogashira growth of a BODIPY‐based CMP shell on the UCNP surface. This strategy affords UC@CMPBDP core@shell nanohybrids with homogeneous polymer coatings, controlled polymer loading, and direct interfacial contact between the UCNP core and the conjugated porous shell (Figure 1D). Moreover, BODIPY‐based CPPs have been previously used as heterogeneous photocatalyst [22, 23, 24], and their absorption wavelengths match the NIR‐triggered emission of the UCNP of our choice [22]. Using this strategy, we obtained monodisperse UC@CMPBDP nanohybrids in which individual UCNPs are covalently linked and uniformly encapsulated within a CPP shell, rather than randomly embedded in a polymer matrix. This architecture maximizes the UCNP/polymer interfacial area while preserving a homogeneous polymer coating with precisely controlled loading. More importantly, it enables a systematic investigation of how both the shell thickness (polymer loading, X wt%) and the UCNP structure (core versus core@shell) influence NIR‐driven photocatalysis. Upon NIR irradiation, the UCNP core converts NIR photons into visible light, which is readily harvested by the surrounding CMPBDP shell to promote the photocatalytic oxidation of *α*‐terpinene. This work establishes a rational synthetic platform for engineering direct UCNP‐CPP interfaces and demonstrates their impact on NIR‐activated photocatalysis.

## 2 Results and Discussion

### 2.1 Nanohybrids Synthesis

First, hexagonal core NaYF_4_: Yb^3+^ (20%), Er^3+^ (2%) and core‐shell NaYF_4_: Yb^3+^ (20%), Er^3+^ (2%)@NaYF_4_ oleate‐capped UCNPs (UC_C_@OA and UC_CS_@OA, respectively) were synthesized following a well‐known protocol (see Sections S3.1–S3.2) [15, 25]. Transmission electron microscopy (TEM) images revealed hexagonal nanoprisms with a size of (20.6 ± 1.5) x (24.7 ± 1.4) nm and (22.8 ± 1.6) x (32.8 ± 1.8) nm, respectively (Figure S1). As previously described, the oleate ligand was removed by using nitrosonium tetrafluoroborate (NOBF_4_) to get UC_C_ or UC_CS_ (see Section S3.3) [26].

Next, UC_C_ or UC_CS_ were coated with 4‐bromobenzenesulfonate (BBS). The successful anchoring of BBS on the surface of the UC_C_ and UC_CS_ NPs was corroborated by the absorption spectra in which the BBS absorption band was clearly observed in the UC_C_@BBS and UC_CS_@BBS nanohybrids (Figure S2). The estimated amount of BBS per milligram of UCNP was 5 and 45 µmol for UC_C_@BBS and UC_CS_@BBS, respectively. TEM images of both nanohybrids confirmed the preservation of the morphology and size of nanoprisms (Figure S3). Subsequently, UC@CMPBDP nanohybrids were prepared from the corresponding BBS‐capped nanoparticles (UC_C_@BBS and UC_CS_@BBS) and the monomers, 1,3,5‐triethynylbenzene and diiodo‐BODIPY (in a 1.1:1 molar ratio), by Pd(0)‐catalysed Sonogashira cross‐coupling reaction (Scheme 1). The polymer loading of 5, 10, and 15 wt.% was controlled by adjusting the molar ratio between UC_C_@BBS or UC_CS_@BBS and the two monomers (refer to the experimental section for further details). We initially prepared four different CMPBDP‐functionalized nanohybrids, with 5, 10, and 15 wt.% polymer loading for UC_CS_ and only the 15 wt.% for UC_C_. Thus, the monomers, [Pd(PPh_3_)_4_] and CuI were dissolved in dimethylformamide (DMF): trimethylamine (Et_3_N) (1:1 v/v). Then, the organic phase was added to a DMF dispersion of UC_C_@BBS (52.5 mg/mL) or UC_CS_@BBS (33.3 mg/mL). The reaction mixtures were then subjected to thermal polymerization according to our previously reported procedure to synthesize the BODIPY‐based conjugated microporous polymer (CMPBDP) [22, 23]. The nanohybrids were isolated by a centrifugation/washing procedure, which removed residual monomers, reagents and polymer without nanoparticles.

**SCHEME 1:**
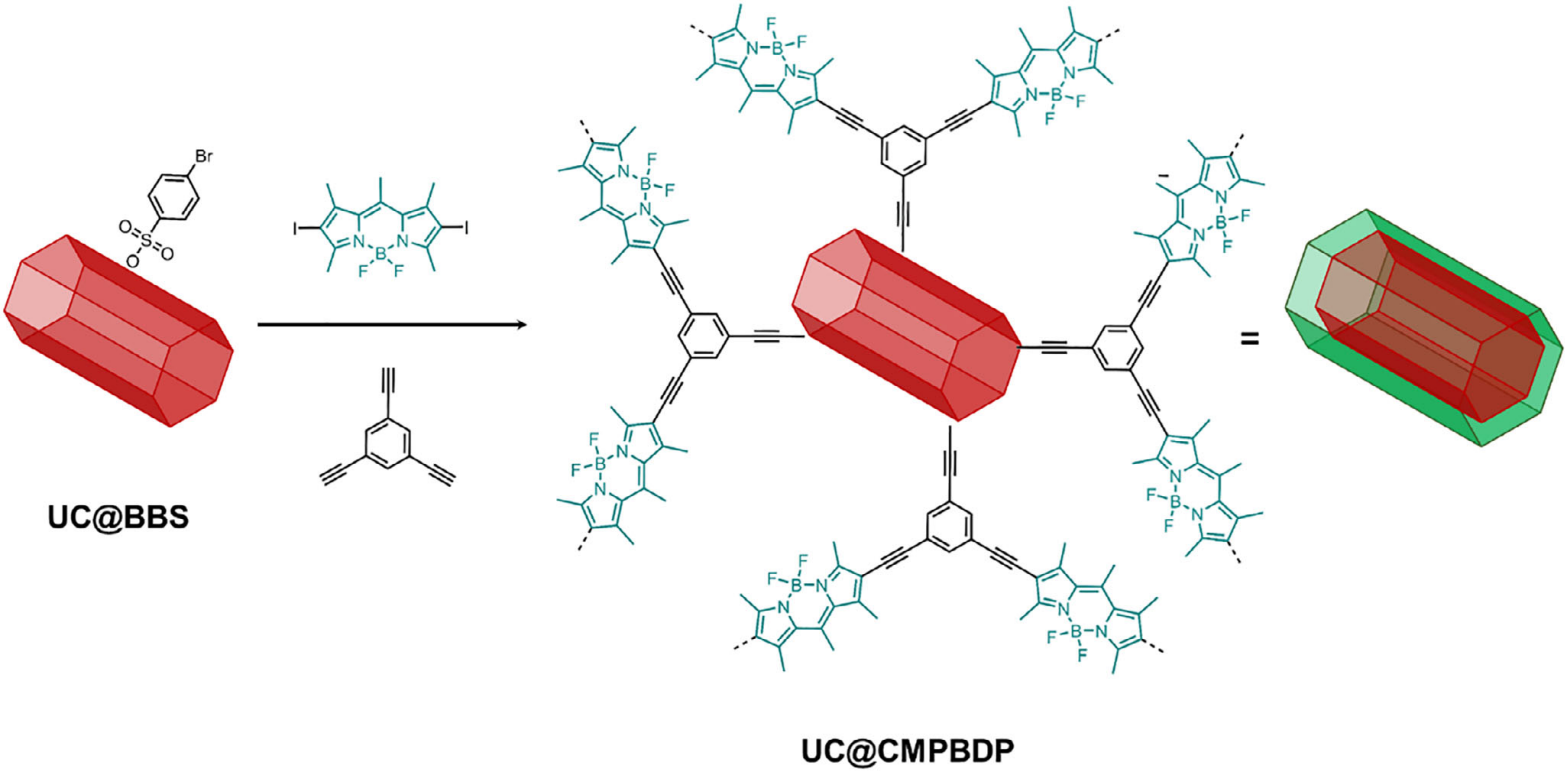
General scheme for the synthesis of UC@CMPBDP by Sonogashira cross‐coupling reaction (The indicated reaction conditions are: CuI/Pd(PPh_3_)_4_ in DMF/Et_3_N and thermal polymerization).

### 2.2 Morphological and Chemical Characterization

^1^H nuclear magnetic resonance (^1^H‐NMR) spectrum of the UC@CMPBDP‐10 nanohybrid corroborated the efficient polymerization of the CMPBDP on the UCNP surface (Figure 2A). Peaks located at 2.6–2.8 ppm, correlate to the methyl groups of BODIPY core and the peak centered at 7.9 ppm is characteristic of the aromatic protons associated to the trifunctionalized phenyl group. In addition, the Attenuated Total Reflectance‐Fourier Transform Infrared (ATR‐FTIR) spectra shown in Figure 2B confirmed the formation of the polymer. This is evidenced by the disappearance of the sharp band corresponding to the terminal alkyne (─C≡C─H) located at ∼3300 cm^−1^, and the presence of the ─C≡C─ stretching band at ca. 2185 cm^−1^. The C═N stretching band, characteristic of BODIPY structure, is also observed at ca. 1650 cm^−1^. Two additional bands at around 2932 and 2852 cm^−1^ were observed, which correspond to the = C─H stretching. Furthermore, the characteristic asymmetric and symmetric S═O stretches from the sulphonate groups, which are part of the linker attached to the nanoparticle's surface, appeared at 1392 and 1092 cm^−1^, respectively. Similar ATR‐FTIR spectra were obtained for UC_CS_@CMPBDP‐X (X = 5 and 15) as it is shown in Figure S4. Both ^1^H‐NMR and ATR‐FTIR data collected for UC_CS_@CMPBDP samples, clearly support the formation of the BODIPY‐based microporous polymer. Their chemical structures perfectly match the one previously reported for the CMPBDP (Figure 2) [22].

**FIGURE 2:**
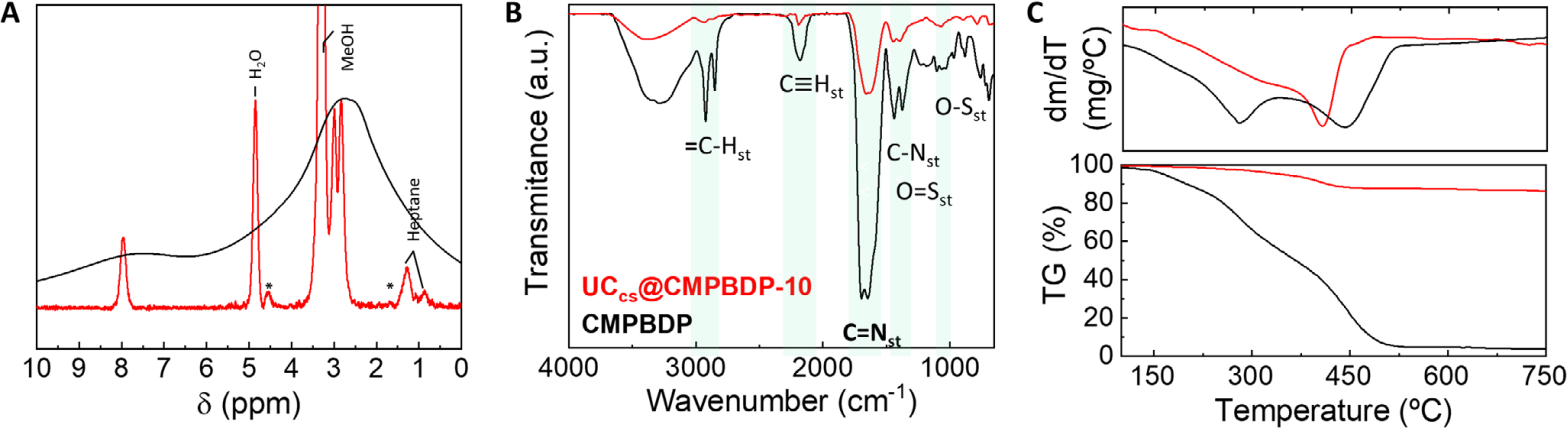
(A) ^1^H‐NMR spectra of UC_CS_@CMPBDP‐10 dispersed in deuterated methanol (red line) versus solid state ^1^H‐NMR of CMPBDP (line black). Note that residual solvent and satellite signals (*) from MeOH are identified. (B) ATR‐FTIR spectra of UCcs@CMPBDP‐10 and CMPBDP. (C) Thermogravimetric analyses (Bottom) and first derivatives (Top) of UC_CS_@CMPBDP‐10 and CMPBDP. Black line: CMPBDP; red line: UC_CS_@CMPBDP‐10.

The different amounts of CMPBDP polymer covalently bound to the UCNP periphery were determined by thermogravimetric analysis (TGA). Thus, the weight contributions of the polymer in the UC_CS_@CMPBDP nanohybrids were found to be ca. 5.5, 11 and 15.3 wt.% and 17% in UC_C_@CMPBDP nanohybrid (Figure S5, Figure 2C). Since the UC_CS_@BBS presents 1 wt.% and UC_C_@BBS presents 1.5 wt.% of functionalization with BSS (Figure S5A,E), the deviation in wt.% of both UC_CS_@CMPBDP and UC_C_@CMPBDP obtained is less than 1 wt.% with respect to the equivalents used in the feed reaction conditions. The main weight losses occurred at slightly higher temperatures to those of CMPBDP [22]. The typical weight loss at ca. 282°C observed for CMPBDP took place at ca. 319°C for UC_CS_@CMPBDP (Figure S5D). This fact suggests a higher thermal stability of the polymer when is attached to the UCNP surface.

Furthermore, the X‐Ray Diffraction (XRD) pattern (Figure S6) of the UC_CS_@CMPBDP‐10 confirmed the preservation of mainly the β‐NaYF_4_ hexagonal phase structure (Joint Committee on Powder Diffraction Standards, JCPDS 16–0334).

The morphology of UC_CS_@CMPBDP‐X was studied by high‐resolution scanning electron microscopy (HR‐SEM) (Figure S7). Additionally, images of the same area taken either by regular FESEM or by backscattered electrons detection mode FESEM (Figure S8) show differences. In backscattered mode only the inner UCNPs are exclusively visualized allowing the measurement of the polymer thickness. The thickness can also be evaluated by TEM (Figure S9). This is exemplified for the UC_CS_@CMPBDP‐10 sample in Figure 3. The hexagonal nanoprisms UCNPs exhibited uniformity, with no significant dimensional changes after their coating with CPP (average size: 24.3 ± 1.6 nm x 31.2 ± 1.9 nm). In the FESEM (Figure 3A) and HR‐SEM (Figure 3C) images, a homogeneous layer surrounding the nanoparticle surface was clearly visible. This layer was absent in the backscattered electron mode (Figure 3B), where only the UCNP core exhibited contrast, confirming that the observed outer layer can be attributed to the CPP capping. Furthermore, TEM images (Figure S9 and Figure 3E) revealed an organic layer surrounding the nanoparticles, consistent with the polymer covalently bound to the UCNP surface. The analysis of these images indicated that the UC_CS_@CPBDP‐10 nanohybrid was surrounded by a thin polymer shell with an approximate thickness of *ca*. 3.7 ± 1.7 nm.

**FIGURE 3:**
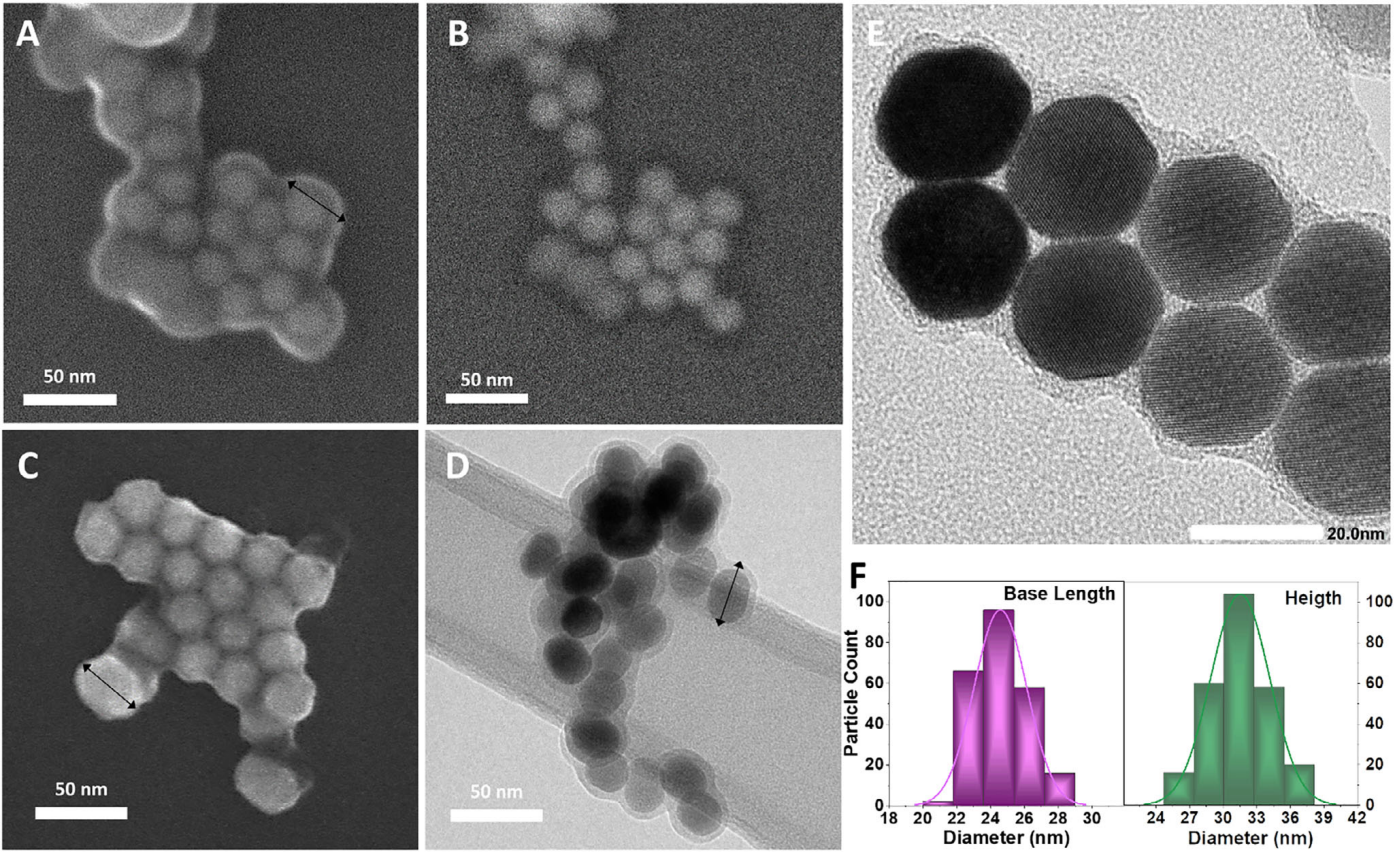
(A) FESEM, (B) FESEM backscattered electrons detection mode, (C) HR‐SEM, (D) and (E) TEM images at different magnifications of hexagonal nanoprisms of UC_CS_@CMPBDP‐10 with a shell thickness of 3.7 ± 1.7 nm. (F) Distribution of particle sizes of UC_CS_@CMPBDP‐10; mean size of 31.2 ± 1.9 nm (height), and a base length of 24.3 ± 1.6 nm. Black arrows indicate the same height.

Furthermore, Energy‐Dispersive X‐Ray(EDX) micrographs confirmed the homogeneous functionalization of the BODIPY based conjugated polymer around the UCNP (Figure 4). Na and F from UCNPs mapping overlap with C mapping from CMPBDP.

**FIGURE 4:**
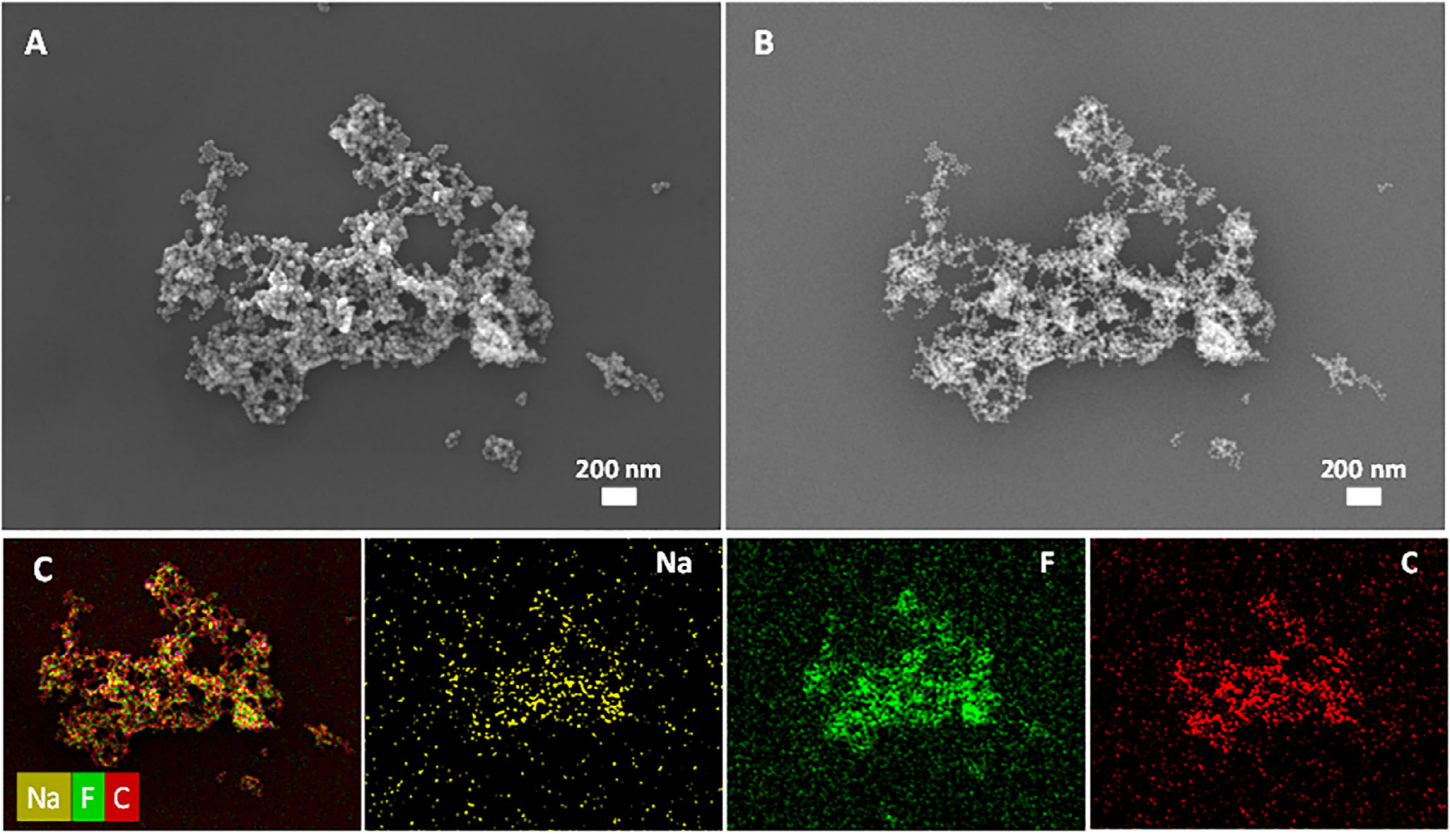
(A) FESEM images and (B) FESEM backscattered electrons detection mode of UC_CS_@CMPBDP‐10. (C) EDX mapping being the first corresponding to the merged image and the next corresponding to Na atoms (yellow), F atoms (green), and C atoms (red) images.

It is important to note that the aggregation of nanohybrids observed in the microscopy images, may result from sample preparation effect, specifically during the drying process. To address this, particle size distributions were measured in DMF dispersion at a concentration of 0.04 mg/mL by using Dynamic Light Scattering (DLS). The solvodynamic sizes of the nanohybrids, averaged over 5 independent measurements and 10 rounds per measurement, are shown in Table S1 and Figure S10. The solvodynamic diameter of UC_CS_@BBS was 47 ± 10 nm, consistent with the value observed in TEM images. Upon functionalization of the nanoparticle periphery with CPP, a notable increase of the solvodynamic diameters was observed, with values of 80 ± 14, 86 ± 15, and 95 ± 17 nm for UC_CS_@CMPBDP‐5, UC_CS_@CMPBDP‐10, and UC_CS_@CMPBDP‐15, respectively. This size increase was attributed to the layer of the polymer and its solvation, which slightly increased with the amount of CPP in the nanohybrid. Also, these values evidenced the absence of aggregation effect of the nanohybrids in dispersion.

The textural properties of the UC_CS_@CMPBDP‐10 nanohybrid were evaluated by N_2_ adsorption isotherm measurement (Figure S11) and its surface area (*ca*. 195 m^2^/g) was calculated using the BET (Brunauer‐Emmett‐Teller) method. The relatively high BET surface area observed, despite the moderate polymer content estimated by TGA, may be related to the different textural organization of the polymer when it is grown directly on the UCNP surface. In contrast to the bulk CPP, which shows a surface area of *ca*. 299 m^2^/g,[22] where the porosity is mainly associated with intrinsic micropores of the polymer network, the interfacial growth of the CPP shell can favor the formation of a more open and nanostructured architecture. The heterogeneous nucleation on the UCNP surface may limit polymer domain aggregation and promote interparticle voids and mesoporous cavities within the polymer shell. This is consistent with the shape of the N_2_ adsorption‐desorption isotherm of the hybrid, which suggests a larger mesoporous contribution compared with the predominantly microporous character of the bulk CPP. Therefore, the BET surface area of the hybrid should not be interpreted only as a weighted contribution of the dense UCNP core and the bulk CPP, but also as the result of a distinct polymer organization induced by surface‐confined growth. Nevertheless, given the relatively low polymer fraction estimated by TGA, the unexpectedly high surface area of the hybrid should be interpreted with caution. Additional contributions from interparticle voids, shell roughness or sample aggregation cannot be completely ruled out.

### 2.3 Photophysical Characterization

The photophysical characterization of the nanohybrids was performed to evaluate the synergistic interactions between CMPBDP and UCNPs. The absorption spectra of the BBS‐functionalized nanohybrids displayed an absorption band below 300 nm, corresponding to the BBS (Figure S2). After surface modification with CMPBDP, a broad absorption band extending from UV region to approximately 700 nm, with a maximum at 320 nm, was observed for all the nanohybrids (Figure 5A). Interestingly, the CMPBDP absorption was significantly lower in comparison to the nanohybrids at the same concentration and exhibited a shoulder at *ca*. 570 nm (Figure S12). This variation was also visibly evident by a color change from purple in the CMPBDP solution to brown in the UC_CS_@CMPBDP dispersion in DMF. As expected, the absorbance increased with increasing polymer loading in the nanohybrid (Figure 5A).

**FIGURE 5:**
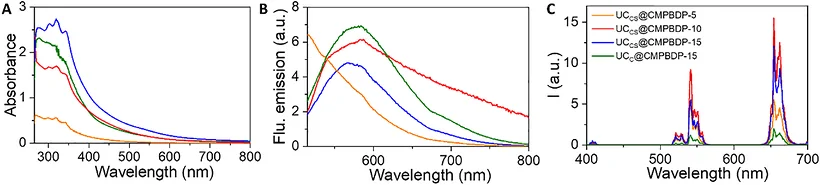
(A) Absorption spectra and emission spectra upon excitation at (B) 500 nm and (C) 980 nm of 0.4 mg/mL nanohybrid dispersions in DMF. Note that the legend inset at C is common for A and B with the next colour code: UC_CS_@CMPBDP‐5 (orange), UC_CS_@CMPBDP‐10 (red), UC_CS_@CMPBDP‐15 (blue), and UC_C_@CMPBDP‐15 (green).

The emission spectra of the CMPBDP in the nanohybrids were compared (Figure 5B). We have previously reported that CMPBDP exhibits two distinct fluorescence emission bands at approximately 515 and 560 nm, whose relative intensities depend on the nature of the solvent [22]. The first probably due to a H‐aggregated emission from *π−π* stacking within the polymer network and the second due to S_0_→S_1_ transition of the BODIPY chromophore. The emission behavior of the CMPBDP changes drastically when present on the periphery of UCNPs. Upon excitation of the hybrid at 500 nm the S_0_→S_1_ emission of transition of the BODIPY chromophore suffered a bathochromic shift to 593 nm as long as the amount of polymer increased. Furthermore, in the hybrid materials the H‐aggregates band did not appear. Interestingly, in the nanohybrid with lower loading of CMPBDP, i.e., UC_CS_@CMPBDP‐5, the emission at 593 nm was negligible. This suggests that, in addition to the conjugation of the polymer to the UCNP surface, the CMPBDP loading also plays a crucial role in the modulation of the emission properties. It is important to note that the CMPBDP emission was very weak in all cases, and its quantum yield could not be measured with our instrumentation.

Regarding the upconverting properties of the nanohybrids, Figure 5C and Figure S13 show the recorded emission spectra upon excitation of the samples with a 980 nm continuous wavelength diode. Here, the typical emissive bands of Er^3+^ cations arising from electronic transitions within its 4f orbitals were observed. The UC emission spectra were corrected considering secondary inner‐filter effects (see SI). The visible emission primarily occurred in the green and red regions of the spectrum. In the green region, two main transitions are observed ^2^H_11/2_→^4^I_15/2_ and ^4^S_3/2_→^4^I_15/2_, resulting in emissions centered at *ca*. 520 nm and 540 nm, respectively. In the red region, the ^4^F_9/2_→^4^I_15/2_ transition generates an emission band around 650 nm. A much less prominent band in the ultraviolet is also shown in Figure 4C, corresponding to the ^2^H_9/2_→^4^I_15/2_ transition.

The intensity of the emissions for core/shell‐based structures were significantly higher than that of core structures, attributed to the surface passivation effect of the shell. Specifically, the initial UC_CS_@BBS nanohybrids exhibited *ca*. 90% greater luminescence than UC_C_@BBS. This was also reflected by the upconversion quantum yield (UCQY) measurements, showing a value of 1.7 and 0.2%, for UC_CS_@BBS and UC_C_@BBS, respectively (see Figure S14). A systematic strong decrease in the emission intensity (*ca*. 90%) was observed in all nanohybrids upon functionalization with CMPBDP, which has also been previously reported in UCNP‐CP composites [25]. This reduction was caused mainly by the strong absorption of CMPBDP, making it excellent energy acceptor in an energy transfer process from the UCNP to the CMPBDP upon NIR excitation. Therefore, the calculated area under the emission spectra of the CMPBDP‐capped nanohybrids, revealed values ten times lower in comparison to its counterparts. The UCQY for the UC_CS_@CMPBDP‐10 was 0.07% while for UC_C_@CMPBDP‐15 was 0.01% which is consistent with the results observed in the recorded steady‐state emission spectra.

Notably, this reduction was more pronounced for the green emission than for the red one, resulting in an increase in the ratio between the integrated red emission band (642–682 nm) and the integrated green emission band (514‐564 nm) as the CMPBDP loading on the nanohybrid increased (Figure S13B). This can be explained by the absorptivity of CMPBDP, which extends from the UV to the VIS region up to *ca*. 600 nm, beyond which its absorption is almost negligible. Consequently, in the 500 to 600 nm spectral region, there is a greater spectral overlap between the UC emission and the CMPBDP absorption. As a result, a more efficient energy transfer from the UCNP to the CMPBDP upon NIR excitation is envisaged in the green than in the red region. No CMPBDP‐sensitized emission was detected upon NIR excitation probably due to the low emission QY of the CMPBDP.

To better understand this quenching process, time‐resolved luminescence measurements were conducted under 980 nm excitation. Tables S2–S6 summarize the estimated lifetimes obtained from the fitted kinetic profiles. As expected, core architecture exhibited significantly shorter lifetimes than those calculated for the core‐shell architectures, owing to the protective effect of the shell, which minimizes non‐radiative deactivation pathways and reduces interactions with the surrounding environment. In core‐based nanohybrids, an evident shortening in lifetimes at 540 nm was observed, corresponding to a quenching efficiency of 13.4% when compared to the UC_C_@BBS nanohybrid. It should be noted that the UCNPs lifetimes were less affected than the upconversion luminescence (UCL) intensity in all the nanohybrids. This observation suggests that the UCL quenching arises from two distinct mechanisms: resonant energy transfer and a re‐absorption effect. Thus, in these nanohybrids the dramatic UCL intensity decrease is mainly attributed to re‐absorption by CMPBDP, caused by the strong absorption of the CMPBDP due to the large number of absorbing π‐conjugated units. This re‐absorption effect reduces the detected emission intensity without affecting the donor emission lifetime, whereas resonant energy transfer accounts for the observed shortening of the emission lifetime. In contrast, the lifetime at 660 nm remained nearly unchanged. As previously explained, the superior spectral overlap at 540 nm than at 660 nm between the UCNP emission and the CMPBDP absorption could justify this observation.

Similarly, in core‐shell nanohybrids, the quenching efficiencies at 540 nm were calculated as 16.0, 5.4, and 16.3% for UC_CS_@CMBDP‐5, UC_CS_@CMBDP‐10, and UC_CS_@CMBDP‐15, respectively. However, the lifetimes at 660 nm displayed a minimal shortening, with reductions below 5% compared to their precursor UC_CS_@BBS. No significant alterations in the UC kinetic behavior were observed under a protective nitrogen atmosphere.

Interestingly, excitation at 980 nm resulted in a long‐lived signal at 580 nm, with a decay lifetime of approximately 200 µs observed for all UC@CMBDP‐X nanohybrids. Notably, this emission was absent in pristine UCNPs, indicating that the 580 nm signal arises from sensitized CMPBDP emission caused by energy transfer from the 980‐nm excited UCNPs. However, due to the inherently low emissive properties of CMPBDP combined with the energy transfer processes, the resulting CMPBDP emission under 980 nm excitation was noisy and not detectable in steady‐state measurements.

Regarding the NIR emission of the nanohybrids upon 980 nm excitation, the nanohybrids displayed the typical Er^3+^ emission band centered at *ca*. 1550 nm (Figure S15). The characteristic singlet oxygen phosphorescence band (ca. 1270) was not detected for any of the nanohybrids under either air or N_2_ atmosphere.

### 2.4 Application of the UC@CMPBDP Nanohybrids as Photocatalysts

Recent studies have shown that BODIPY‐based CPP materials exhibit photocurrent generation upon light exposure, demonstrating their ability to respond through both electrons and holes. This behavior highlights BODIPY based CPPs’ dual capacity for oxidative and reductive processes, making them highly adaptable for photocatalytic applications. Light irradiation of CPPs undergo the generation of Reactive Oxygen Species (ROS) by the electrons in the conduction band. Furthermore, BODIPY‐based CPPs have demonstrated photocatalytic activity via singlet oxygen [22], and, when combined with TiO_2_, they are the responsible of the oxidative pathway in the hydrogen production from water using methanol as sacrificial agent [23]. Moreover, miniaturization of the CPPs to the nanoscale has been shown to enhance the overall photocatalytic activity of the system [24].

We envisaged that the controlled growing of CMPBDP around the UCNP periphery will endorse the nanohybrid with ability to undergo chemical reaction upon NIR exposure, due to the energy transfer from the UCNPs to the conduction band of CMPBDP. Thus, to assess the photochemical reactivity of our nanohybrids under NIR light activation, α‐terpinene photooxidation was selected as a model reaction (Scheme 2). In this process, α‐terpinene, a natural terpenoid, reacts with ROS generated by the photocatalyst through several oxidation pathways: i) oxidation of the methyl group to afford benzyl alcohol (**1**), aldehyde (**2**) and, even, carboxylic acid (**3**); ii) Diels‐Alder cycloaddition between O_2_ and the diene to form ascaridole, which can evolve into linear aldehydes (**4** and **5**) and carboxylic acids (**6** and **7**), Furthermore, products arising from thermal processes can be observed, including p‐cymene (as result of a reduction pathway) andβ‐ and γ‐ terpinene (isomerization pathway) [27].

**SCHEME 2:**
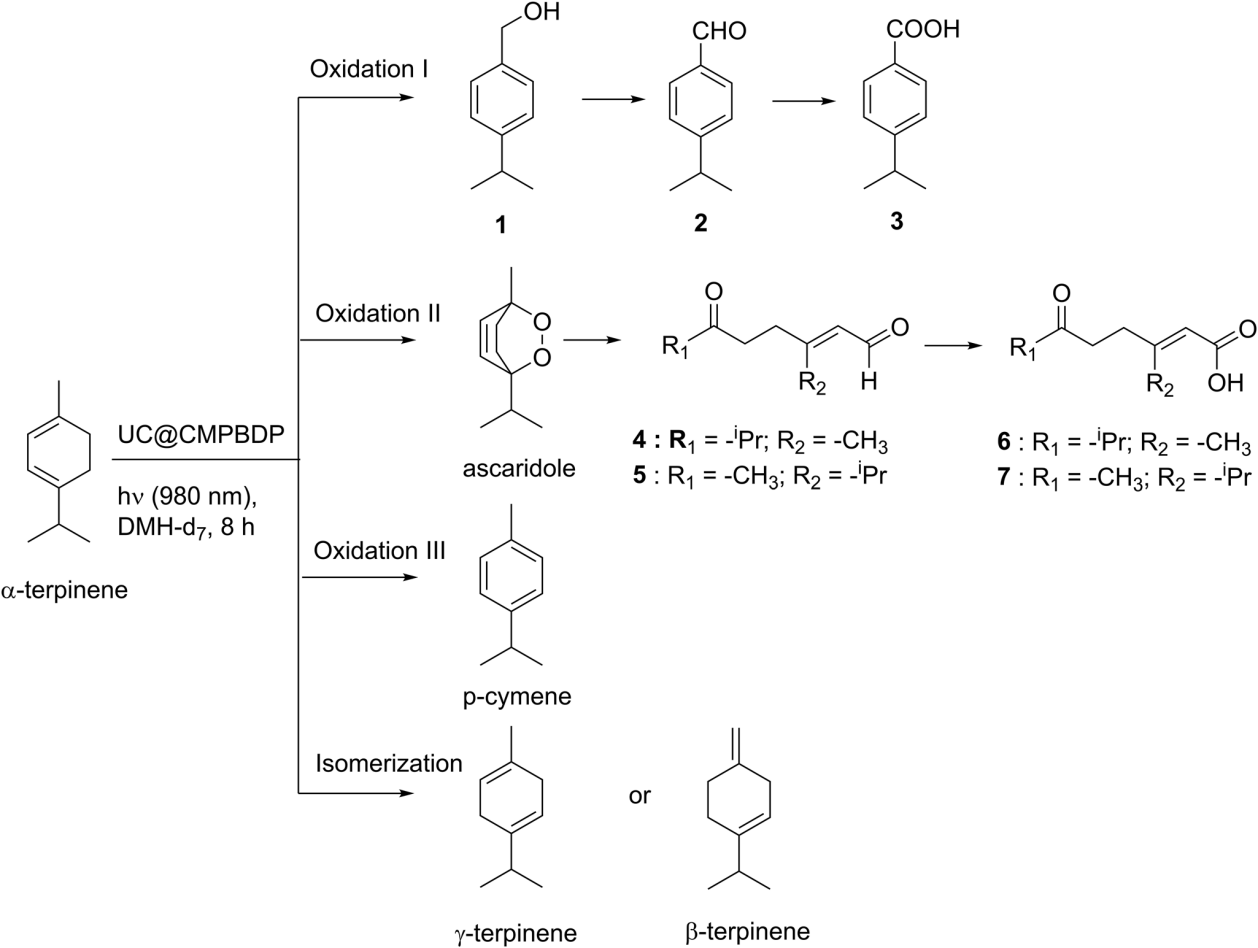
Photooxidation and other thermal reaction pathways of α‐terpinene upon irradiation of UC@CMPBDP nanohybrids (λ_exc_ = 980 nm) under oxygen.

To evaluate the photocatalytic performance of the UC@CMPBDP nanohybrids, a 200 µL dispersion containing α‐terpinene (0.06 M) and the UC@CMPBDP nanohybrids in DMF‐d_7_ was irradiated at 980 nm for 8 h under oxygen atmosphere. Following irradiation, the reaction mixture was centrifuged to separate the nanohybrids, and the supernatant was analyzed by ^1^H‐NMR. Reaction yields were determined by integrating the corresponding ^1^H‐NMR signals (see Supporting Information for details). The results obtained for each UC@CMPBDP nanohybrid are displayed in Table 1. Further washing of UC@CMPBDP nanohybrids with DMF proved that there were no products adsorbed on the CMPBDP surface.

**TABLE 1:** Photocatalytic activity.

						Yield (%)					
Entry	PC	Light source	[PC] (mg/mL)	Overall Conversion (%)	Photoproduct Conversion (%)	Ascaridole	*p*‐cymene	*γ*‐terpinene	*β*‐terpinene	1^b^	Aldehydes
1	UC_C_@CMPBDP‐15	NIR	21	52	13	25	26.9	48.1	0	0	0
2	UC_C_@CMPBDP‐15	NIR	25	49	5	10.2	28.6	24.5	36.7	0	0
3	UC_C_@CMPBDP‐15	NIR	35	56	22	17.9	12.5	48.2	0	21.4	0
4	CMPBDP	NIR	3.5	38	0	0	68.4	31.6	0	0	0
5	CMPBDP	SS^a^	3.5	100	75	59.5	10.5	14.5	0	0	15.5
6	UC_CS_@CMPBDP‐5^c^	NIR	35	59.0	42	39.0	9.3	19.5	0	0	32.2
7	UC_CS_@CMPBDP‐10	NIR	35	58.9	26.9	31.6	27.2	27.2	0	0	14
8	UC_CS_@CMPBDP‐15	NIR	35	45.3	24	37.5	24.3	22.7	0	0	15.5
9	UC_CS_@CMPBDP‐10	SS	35	94.5	74.0	64.6	8.8	12.9	0	0	13.8
10	UC_CS_@CMPBDP‐5	None	35	14	0	0	53	47	0	0	0

^a^ SS = Solar simulator.

^b^ 4‐isopropylylbenzyl alcohol.

^c^ reaction volume: 180 µL.

First, the effect of the photocatalyst concentration on the reaction yield was evaluated. Thus, irradiations were carried out using UC_C_@CMPBDP‐15 nanohybrid concentration of 21, 25, and 35 mg/mL (entries 1–3 in Table 1). The results showed similar α‐terpinene conversion for all assays; however, higher photocatalyst concentration led to an increase in the conversion to photoproducts (*ca*. 22%). Based on these findings, we selected a concentration of 35 mg/mL for subsequent experiments.

A series of control experiments were carried out by irradiating α‐terpinene in the presence of CMPBDP and in the absence of nanohybrids, employing both NIR irradiation and solar simulator. When NIR light was employed, the conversion remained low (*ca*. 38%) and no significant formation of photoproduct, ascaridole or aldehydes, was detected, whereas *p*‐cymene (68%) and γ‐terpinene (31.6%) were formed (entry 4, in Table 1). In contrast, irradiation with a solar simulator resulted in complete conversion, with the photoproduct conversion close to 75%. Under this conditions, substantial formation of ascaridole (59.5% yield) was observed, together with aldehydes in 15.5% yield (entry 5, in Table 1). These results indicate that the oxidation products observed in experiments with nanohybrids arise exclusively from the selective oxidation of α‐terpinene by photogenerated ROS, produced upon NIR excitation of the UCNPs and subsequent energy transfer to CMPBDP. In contrast, the formation of p‐cymene and the isomerization product predominantly proceeds through thermal pathways.

Later, the NIR‐triggered photooxidation activity of all core‐shell nanohybrids was assessed. While the overall conversion was slightly higher than that observed in the core nanohybrid, a much morepronounced increase in the conversion to photoproducts was observed. Thus, conversions to photoproducts of 42%, 26.9%, awere obtained for UC_CS_@CMPBDP‐5, UC_CS_@CMPBDP‐11, and UC_CS_@CMPBDP‐15, respectively (entriess 6–8, in Table 1). As expected, and based on these results, the core‐shell nanohybrids are more photoactive than core nanohybrid and, also, the nanohybrid with the thinniest polymer coating, UC_CS_@CMPBDP‐5, shows the best results. Furthermore, the products selectivity changes if we compare core and core‐shell nanohybrids. The formation of ascaridole and the aldehydes was be favored for the core‐shell nanohybrids while the isomerization products were favored for the core nanohybrids. This can be attributed not only to the enhancement of the luminescence intensity but also to the lengthening of the luminescence lifetimes. This lengthening of excited states duration allows for a greater likelihood of effective energy transfer from the UCNP to the CMPBDP under NIR excitation. Consequently, the amount of the available photogenerated ROS increased and with this, the reaction yield to oxidation products.

Our strategy proved to be significantly more efficient for NIR‐triggered photooxidation compared to previously reported UCNP‐CPP composites, where the reaction yield was ca. 15% [15]. In the previous work, the nanohybrids consisted of core UCNPs and AuNPs for plasmon‐enhanced UCL, with both NPs either linked or adsorbed onto the polymeric composite, respectively. The adsorption of AuNPs within the polymer pores likely reduced the oxygen diffusion into the porous structure, thereby limiting the formation of ROS. In contrast, our approach enables precise control over the growth of a polymeric shell around the UCNPs. This miniaturization to the nanoscale enhances the surface‐to‐volume ration, increasing the availability of the CMPBDP surface to interact with oxygen. As a result, the reaction yield was considerably improved.

More interestingly, we also evaluated the recyclability of our UC@CMPBDP photocatalysts. Thus, once the photoreaction finalized, the nanohybrids were allowed to precipitate by centrifugation and, subsequently, re‐dispersed in DMF. Upon addition of a freshly prepared α‐terpinene solution, the reaction was excited with the 980 nm laser diode for 8 h. After, the centrifugation, the reaction mixture was analyzed by ^1^H‐NMR. The cycle was repeated 5 times for UC_CS_@CMPBDPs. The results are shown in Figure 6 and Tables S9 and S10.

**FIGURE 6:**
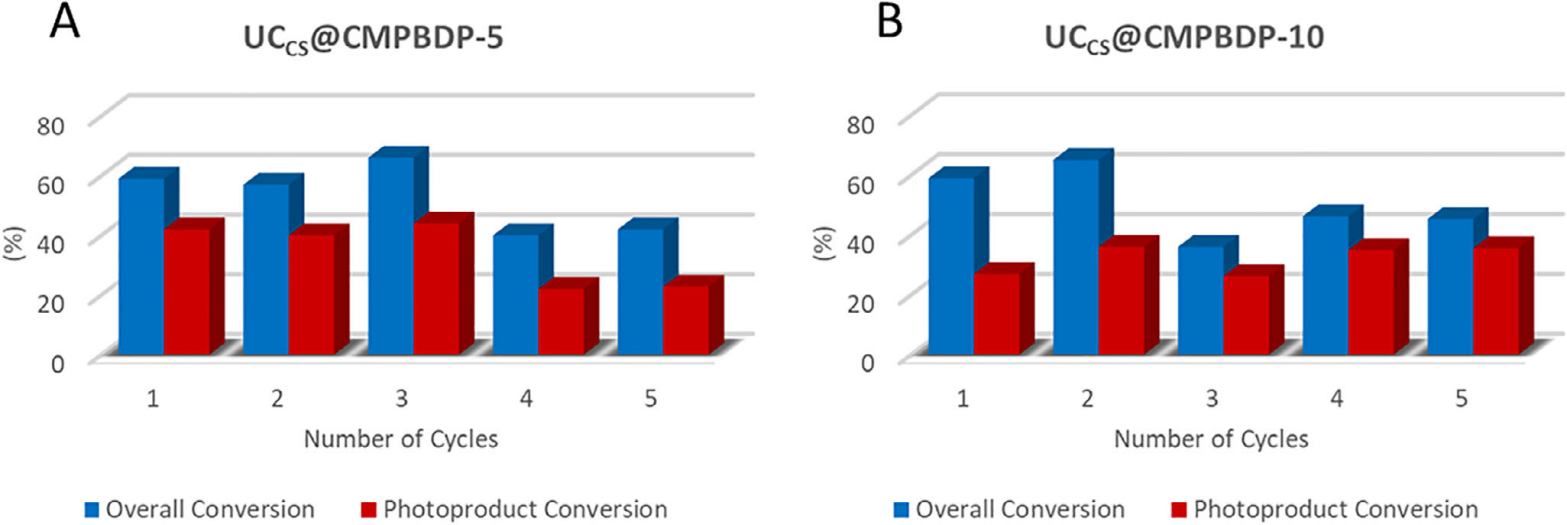
Recycling experiments of UC_CS_@CMPBDP‐5 and UC_CS_@CMPBDP‐10 nanohybrids.

UC_CS_@CMPBDP‐5 was successfully reused as photocatalyst up to three times (Figure 6A). After that, both the overall conversion and photoproduct conversion fall. At this point we wonder if the UCCS@CMPBDP‐10 with large CMPBDP thickness would have a better performance. UC_CS_@CMPBDP‐10 nanohybrids displayed an exceptional reusability up to five consecutive photooxidation reactions (Figure 6B). In spite that the overall conversion decrease in the third cycle the photoproduct conversion was stable in all the cycles. The superior behavior of the UC_CS_@CMPBDP‐10 nanohybrids can be explained by the improved UCQY of the nanohybrid which results from achieving an optimal balance between the amount of CMPBDP and the UC emissive properties of the UCNP. This balance enhances the generation of a higher amount of ROS upon NIR excitation, and consequently the photocatalytic performance.

The exceptional recyclability demonstrated by these nanohybrids addresses a major limitation of previous UCNP‐based photocatalysts, where reusability was compromised by the leaching of the photosensitizer from the UCNP surface [5]. Thus, the covalent linking of the polymeric shell together with its porosity ensures a more robust strategy that effectively prevents the loss of photocatalytic activity over multiple cycles.

Lastly, the photocatalytic activity of the nanohybrids was evaluated using a solar simulator for the excitation of UC_CS_@CMPBDP‐10. The overall reaction conversion increased drastically to 94% with more than 74% of conversion to photoproducts, ascaridole being the major product (64% yield) (entry 9, Table 1), This result aligns with expectation, given that the sample was irradiated across a broad spectral range from 250 to 1800 nm. Consequently, the photocatalytic activity of the nanohybrid was due to the direct excitation of the CMPBDP within its wide absorption range, reaching a complete consumption of the α‐terpinene. Also, it is important to know if α‐terpinene indergopes any reaction in the presence of the nanohybrid and in the absence of light. The most photoactive hybrid UC_CS_@CMPBDP‐5 was tested otherwise indentical reaction conditions in the dark and, a 14% of conversion to *p*‐cymene and the isomerization product was observed. No photoproducts were detected (entry 10, Table 1), confirming that only the excitation of the polymer led to photoproducts.

## 3 Conclusions

Despite the notable advantages of UCNPs for NIR‐activated photocatalytic applications, challenges such as photosensitizer leaching have limited the performance and reusability of UCNP‐based photocatalysts. Here, we present an innovative strategy for enhancing the efficiency of NIR‐triggered photooxidations by encapsulating the UCNP surface within a robust shell of a BODIPY‐based CPP. Our approach addresses critical limitations of previous designs, achieving a significant improvement in the reaction yield of up to 42% under NIR irradiation.

The secret behind this success lies in the precise control over the growth of a polymeric shell of CMPBDP around the UCNPs surface, thanks to advanced synthetic protocols. Three key parameters were essential for controling the polymer coating: (i) prefunctionalization of UCNPs with an active moiety (i.e., BSS) capable of participating in the C─C cross coupling reaction of our choice (Sonogashira reaction); (ii) control of the amount of monomer used in the feed; (iii) an approrpiate work‐up to isolate the UC@CPP nanohybrid from free polymer and vice versa. This strategy not only ensures a uniform distribution of CMPBDP around the UCNP but also maximizes the exposure of CMPBDP to oxygen, promoting efficient oxygen diffusion through the pores of the polymeric shell. Furthermore, the core‐shell nanohybrids based on core‐shell UCNPs demonstrated a photocatalytic efficiency that was twice as high as that of their core‐only counterparts, highlighting the significant advantage of the NaYF_4_ shell architecture in enhancing reaction performance.

When the photooxygenation was carried out under simulated sunlight, using excitation wavelengths from UV to NIR, a reaction yield of ca. 65% was obtained. Moreover, the developed UCNP‐based photocatalysts exhibited excellent recyclability, keeping their photoactivity over at least five reaction cycles. Thus, evidencing their strong potential in greener and more sustainable photocatalytic processes.

Overall, this work highlights the advantages of controlled CPP fabrication on the UCNP surface for achieving nanoscale precision and demonstrates the potential of UC@CMPBDP nanohybrids as efficient and recyclable photocatalytic systems. Although the oxidation yields obtained in this study are moderate, the successful preparation of these materials provides a basis for further optimization. Their performance is also currently being explored in a broader range of photodynamic and photocatalytic applications.

## Conflicts of Interest

The authors declare no conflicts of interest.

## Supporting information




**Supporting File**: smll75481‐sup‐0001‐SuppMat.docx.

## Data Availability

The data that supports the findings of this study are available in the supplementary material of this article.

## References

[1] X. Liu , C.‐H. Yan , and J. A. Capobianco , “Photon Upconversion Nanomaterials,” Chemical Society Reviews 44 (2015): 1299–1301, 10.1039/C5CS90009C. 25716767

[2] B. Zhou , B. Shi , D. Jin , and X. Liu , “Controlling Upconversion Nanocrystals for Emerging Applications,” Nature Nanotechnology 10 (2015): 924–936, 10.1038/nnano.2015.251. 26530022

[3] L. Francés‐Soriano , M. González‐Béjar , and J. Pérez‐Prieto “Synergistic Effects in Organic‐Coated Upconversion Nanoparticles,” in Upconverting Nanomaterials: Perspectives, Synthesis, and Applications (ed. C. Altavilla ) (CRC Press, 2016), pp. 101–138, 10.1201/9781315371535.

[4] J. Ferrera‐González , M. González‐Béjar , and J. Pérez‐Prieto "Functional Nanohybrids Based on Dyes and Upconversion Nanoparticles" in Dyes and Photoactive Molecules in Microporous Systems, (ed. V. Martínez‐Martínez and F. L. Arbeloa ) (Springer International Publishing, 2020), 371–396, 10.1007/430_2020_60.

[5] L. Francés‐Soriano , D. Bellezza , J. Ferrera‐González , M. González‐Béjar , and J. Pérez‐Prieto , “NIR‐Triggered Photooxygenation of α‐Terpinene With Upconversion Nanohybrids,” Nanoscale Advances 6 (2024): 5889–5896, 10.1039/D4NA00528G. PMC11440474 39355838

[6] J. Ferrera‐González , M. González‐Béjar , and J. Pérez‐Prieto , “Synergistic or Antagonistic Effect of Lanthanides on Rose Bengal Photophysics in Upconversion nanohybrids?,” Nanoscale 15 (2023): 19792–19800, 10.1039/D3NR03774F. 38050867

[7] I. Pérez‐Herráez , J. Ferrera‐González , E. Zaballos‐García , M. González‐Béjar , and J. Pérez‐Prieto , “Raspberry‐Like Nanoheterostructures Comprising Glutathione‐Capped Gold Nanoclusters Grown on the Lanthanide Nanoparticle Surface,” Chemistry of Materials 36 (2024): 4426–4436, 10.1021/acs.chemmater.3c03333. PMC11099914 38764750

[8] J. S. M. Lee and A. I. Cooper , “Advances in Conjugated Microporous Polymers,” Chemical Reviews 120 (2020): 2171–2214, 10.1021/acs.chemrev.9b00399. PMC7145355 31990527

[9] H. Ali , O. Iqbal , M. Sadiq , et al., “Novel Advancements in Synthesis, Modulation, and Potential Applications of Conjugated Microporous Polymer‐Based Materials,” Nano Materials Science 8 (2026): 117–174, 10.1016/j.nanoms.2024.08.008.

[10] M. Barawi , L. Collado , M. Gomez‐Mendoza , F. E. Oropeza , M. Liras , and V. A. de la Peña O'Shea , “Conjugated Porous Polymers: Ground‐Breaking Materials for Solar Energy Conversion,” Advanced Energy Materials 11 (2021): 2101530, 10.1002/aenm.202101530.

[11] M. González‐Béjar , “Photoactive Hybrid Materials based on Conjugated Porous Polymers and Inorganic Nanoparticles,” Advanced Photonics Research 2 (2021): 2100060, 10.1002/adpr.202100060.

[12] M. Liras , M. Barawi , and V. A. de la Peña O'Shea , “Hybrid Materials Based on Conjugated Polymers and Inorganic Semiconductors as Photocatalysts: From Environmental to Energy Applications,” Chemical Society Reviews 48 (2019): 5454–5487, 10.1039/C9CS00377K. 31608912

[13] P. Mukherjee , S. Guha , G. Das , A. Samui , and S. Kumar , “NIR Light‐Activated Upconversion POP Nanofiber Composite ; An Effective Carrier for Targeted Photodynamic Therapy and Drug Delivery,” Journal of Photochemistry and Photobiology A: Chemistry 443 (2023): 114907, 10.1016/j.jphotochem.2023.114907.

[14] P. Mukherjee , S. Guha , A. Ghosh , K. Kar , G. Das , and S. K. Sahu , “Porous Organic Polymer‐Based Nanocomposites for Hypoxia Relieving and Enhanced Chemotherapy in Hepatocellular Carcinoma,” ACS Applied Bio Materials 7 (2024): 6138–6151, 10.1021/acsabm.4c00723. 39177187

[15] S. Jaiswal , A. Giri , D. Mandal , M. Sarkar , and A. Patra , “UV‐to‐NIR Harvesting Conjugated Porous Polymer Nanocomposite: Upconversion and Plasmon Expedited Thioether Photooxidation,” Angewandte Chemie International Edition 62 (2023): 202312910, 10.1002/anie.202312910. 37823846

[16] D. W. Kim , P. Wrede , A. Rodríguez‐Camargo , et al., “Upconversion Nanoparticle‐Covalent Organic Framework Core–shell Particles as Therapeutic Microrobots Trackable With Optoacoustic Imaging,” Advanced Materials 37 (2025): 2418425, 10.1002/adma.202418425. PMC12691905 40052638

[17] J. Liu , Q. Chen , Y.‐N. Sun , M.‐Y. Xu , W. Liu , and B.‐H. Han , “Gold Nanoparticles Encapsulated in Hierarchical Porous Polycarbazole: Preparation and Application in Catalytic Reduction,” RSC Advances 6 (2016): 48543–48549, 10.1039/c6ra04515d.

[18] S. Bhattacharyya , D. Samanta , S. Roy , V. P. Haveri Radhakantha , and T. K. Maji , “In Situ Stabilization of Au and Co Nanoparticles in a Redox‐Active Conjugated Microporous Polymer Matrix: Facile Heterogeneous Catalysis and Electrocatalytic Oxygen Reduction Reaction Activity,” ACS Applied Materials & Interfaces 11 (2019): 5455–5461, 10.1021/acsami.8b20610. 30628430

[19] P. Wang , F. Zhou , K. Guan , et al., “In Vivo Therapeutic Response Monitoring by a Self‐ Reporting Upconverting Covalent Organic Framework Nanoplatform,” Chemical Science 11 (2020): 1299–1306, 10.1039/C9SC04875H. PMC8148386 34123254

[20] L. Chen , W. Wang , J. Tian , et al., “Imparting Multi‐Functionality to Covalent Organic Framework Nanoparticles by the Dual‐Ligand Assistant Encapsulation Strategy,” Nature Communications 12 (2021): 4556, 10.1038/s41467-021-24838-7. PMC8316466 34315880

[21] D. Mendez‐Gonzalez , V. T. Vera , I. Z. Gutierrez , et al., “Upconverting Nanoparticles in Aqueous Media: Not a Dead‐End Road. Avoiding Degradation by Using Hydrophobic Polymer Shells,” Small 18 (2022): 2105652, 10.1002/smll.202105652. 34897995

[22] M. Liras , M. Iglesias , and F. Sánchez , “Conjugated Microporous Polymers Incorporating BODIPY Moieties as Light‐Emitting Materials and Recyclable Visible‐Light Photocatalysts,” Macromolecules 49 (2016): 1666–1673, 10.1021/acs.macromol.5b02511.

[23] L. Collado , T. Naranjo , M. Gomez‐Mendoza , et al., “Conjugated Porous Polymers Based on BODIPY and BOPHY Dyes in Hybrid Heterojunctions for Artificial Photosynthesis,” Advanced Functional Materials 31 (2021): 2105384, 10.1002/adfm.202105384.

[24] S. Palenzuela‐Rebella , T. Naranjo , M. Gomez‐Mendoza , et al., “High‐Performance Microfluidic Techniques Toward Nanostructuration of BODIPY and BOPHY Based CPPs Hybrid Photocatalyst for Hydrogen Production,” Advanced Functional Materials 34 (2024): 2403778, 10.1002/adfm.202403778.

[25] X. Guo , L. Li , W. Jia , et al., “Composite Nanomaterials of Conjugated Polymers and Upconversion Nanoparticles for NIR‐Triggered Photodynamic/Photothermal Synergistic Cancer Therapy,” ACS Applied Materials Interfaces 16 (2024): 19926–19936, 10.1021/acsami.3c12553. 37975246

[26] A. Dong , X. Ye , J. Chen , et al., “A Generalized Ligand-Exchange Strategy Enabling Sequential Surface Functionalization of Colloidal Nanocrystals,” Journal of the American Chemical Society 133 (2011): 998, 10.1021/jal08948z. 21175183

[27] C. Mendoza , N. Emmanuel , C. A. Páez , L. Dreesen , J. C. M. Monbaliu , and B. Heinrichs , “Improving Continuous Flow Singlet Oxygen Photooxygenation Reactions With Functionalized Mesoporous Silica Nanoparticles,” ChemPhotoChem 2 (2018): 890–897, 10.1002/cptc.201800148.

