# Supplementary material for: Direct Growth of BODIPY‐Based Conjugated Porous Polymer Shell on Upconversion Nanoparticles for NIR‐Driven Photocatalysis
Source: Small. 2026 Sep 10;22(63):e75481. doi: 10.1002/smll.75481 (PMC13755997; doi:10.1002/smll.75481)
Supplement: Supplementary file 1 [file SMLL-22-e75481-s001.docx]

**Direct Growth of BODIPY-based Conjugated Porous Polymer shell on Upconversion Nanoparticles for NIR-driven Photocatalysis**

Supporting Information

Teresa Naranjo,^[a,b]^ Laura Francés-Soriano, ^[c,d]^ Delia Bellezza,^[c]^ Julia Pérez-Prieto,^[c]^ Víctor A. de la Peña O´Shea,^[a]^ Marta Liras,*^[a]^ María González-Béjar*^[c]^

Dedicated to the memory of Professor Miguel Ángel Miranda.

[a]	Dr. T. Naranjo, Dr. V. A. de la Peña O´Shea and Dr. M. Liras
Photoactivated Processes Unit
IMDEA Energía
Av. Ramón de la Sagra, 3 28935 Móstoles Madrid (Spain)
E-mail: [marta.liras@imdea.org](mailto:marta.liras@imdea.org)

[b]	Current address: Dr. T. Naranjo

IMDEA Nanoscience

C/ Faraday, 9, 28049 Fuencarral-El Pardo Madrid (Spain)

[c]	Dr. L. Francés-Soriano, Dr. Delia Bellezza, Dr. J. Pérez-Prieto and Dr. M. González-Béjar
Instituto de Ciencia Molecular (ICMol)/Departamento de Química Orgánica
Universitat de València
C/ Catedrático José Beltrán, 2, 46980 Paterna Valencia (Spain)

E-mail: [maria.gonzalez@uv.es](mailto:maria.gonzalez@uv.es)

[d]	Current address: Dr. L. Francés-Soriano
Grupo de Procesos de Oxidación Avanzada, Departamento de Ingeniería Textil y Papelera.

Universitat Politècnica de València
Pl. Ferrándiz Carbonell, s/n, 03801, Alcoi (Spain)

**INDEX**

[1.	General Information	3](#_Toc234656301)

[2.	Synthesis of Monomer	6](#_Toc234656302)

[2.1.	Synthesis of 4,4-difluoro-2,6-diiodo-1,3,5,7-tetramethyl-4-bora-3a,4a-diaza-s-indacene (I-BODIPY).	6](#_Toc234656303)

[3.	Synthesis of tetrafluoroborate-stabilized upconverting nanoparticles.	7](#_Toc234656304)

[3.1.	Synthesis of oleate-capped core NaYF_4_: Yb^3+^, Er^3+^ NPs (UC_C_@OA)	7](#_Toc234656305)

[3.2.	Synthesis of oleate-capped core/shell NaYF_4_: Yb^3+^, Er^3+^@NaYF_4_ NPs (UC_CS_@OA)	7](#_Toc234656306)

[3.3.	Synthesis of UC_C_ or UC_CS_	7](#_Toc234656307)

[4.	Synthesis of UC@BBS	8](#_Toc234656308)

[5.	Synthesis of UC@CMPBDP	8](#_Toc234656309)

[6.	Nanohybrids Characterization	9](#_Toc234656310)

[Figure S1. TEM images of UC_C_ and UC_CS_	9](#_Toc234656311)

[Figure S2. Absorption spectra of UCc@BBS and UC_CS_@BBS	9](#_Toc234656312)

[Figure S3. TEM images of UC_C_@BBS and UC_CS_@BBS	10](#_Toc234656313)

[Figure S4. FTIR-ATR spectra of UC_CS_@CMPBDP	10](#_Toc234656314)

[Figure S5. Thermogravimetric analyses of UC_CS_@BSS and UC_CS_@CMPBDP	11](#_Toc234656315)

[Figure S6. Powder XRD spectrum of UC_CS_@CMPBDP-10	12](#_Toc234656316)

[Figure S7*.*HR-SEM images of UC_CS_@CMPBDP-10	13](#_Toc234656317)

[Figure S8. UC_CS_@CMPBDP-X images taken by FESEM	14](#_Toc234656318)

[Figure S9. TEM images	15](#_Toc234656319)

[Table S1. Solvodynamic sizes of the UCNP nanohybrids from DLS	16](#_Toc234656320)

[Figure S10. Solvodynamic particle size distribution	16](#_Toc234656321)

[Figure S11. Nitrogen sorption isotherms of UC_CS_@CMPBDP-10 at 77 K	17](#_Toc234656321)

[Figure S12. Absorption and emission spectra of CMPBDP	18](#_Toc234656322)

[Figure S13. Emission spectra (λ_exc_= 980 nm) of CMPBDP, UC_CS_@BSS and UC_CS_@CMPBDP	18](#_Toc234656323)

[Figure S14. Upconversion quantum yield (λ_exc_= 980 nm) in DMF	19](#_Toc234656324)

[Figure S15. Kinetic profiles of the nanohybrids in DMF	19](#_Toc234656325)

[Table S2. Lifetimes of the nanohybrids (λ_exc_=980 nm, λ_em_=540 nm) in air	20](#_Toc234656326)

[Table S3. Lifetimes of the nanohybrids (λ_exc_=980 nm, λ_em_=540 nm) in N_2_.	20](#_Toc234656327)

[Figure S16. Kinetic profiles of the nanohybrids (λ_exc_= 980 nm, λ_em_=580 nm) in DMF	21](#_Toc234656328)

[Table S4. Lifetimes of the nanohybrids (λ_exc_=980 nm, λ_em_=580 nm) in N_2_.	21](#_Toc234656329)

[Figure S17. Kinetic profiles of the nanohybrids (λ_exc_= 980 nm, λ_em_=660 nm) in DMF	21](#_Toc234656330)

[Table S5. Lifetimes of the nanohybrids (λ_exc_= 980 nm, λ_em_=660 nm) in air	22](#_Toc234656331)

[Table S6. Lifetimes of the nanohybrids (λ_exc_=980 nm, λ_em_=660 nm) in N_2_.	22](#_Toc234656332)

[Figure S18. NIR emission of the nanohybrids (λ_exc_= 980 nm, λ_em_=1550 nm) in air	23](#_Toc234656333)

[Table S7. Lifetimes of the nanohybrids (λ_exc_=980 nm, λ_em_=1550 nm) in air	23](#_Toc234656334)

[Table S8. Lifetimes of the nanohybrids (λ_exc_=980 nm, λ_em_=1550 nm) in N_2_	23](#_Toc234656335)

[7.	Inner-filter effect	24](#_Toc234656336)

[8.	Photocatalytic reaction	25](#_Toc234656337)

[9.	Recyclability of the photocatalysts	25](#_Toc234656338)

[10.	Solar simulator	25](#_Toc234656339)

[11.	Calculation of reaction yields	25](#_Toc234656340)

[11.1.	^1^H-NMR characterization	26](#_Toc234656341)

[11.2.	Recyclability assays	27](#_Toc234656342)

[12.	References	29](#_Toc234656343)

# 1. General Information

All reagents, bases, catalysts and solvents were commercially available and were used without prior purification.

α-terpinene (Thermo Scientific Chemicals, >90 %) was previously purified by column chromatography on Merck silica gel 60 (230-400 mesh) with n-hexane (Scharlab, 96% reagent grade) as eluent to obtain > 97% purity. The separation was monitored using TLC on silica gel plated aluminium sheets and KMnO_4_ basic solution was used for detection.

**Fourier-transform infrared spectroscopy (FTIR)** spectra are reported in terms of the wavenumber of absorption (cm^-1^) and were registered on a Thermo-Scientific model Nicolet 6700 spectrometer.

**Nuclear magnetic resonance (NMR)**. For the hybrid characterization: Nuclear magnetic resonance (NMR) spectra were recorded with a Varian Mercury AS400 spectrometer (Larmor frequencies of 100 and 400 MHz for ^13^C and ^1^H, respectively) for liquids and a Bruker AVIII/HD spectrometer (Larmor frequencies of 100 MHz for ^1^H) for solids. 4-mm MAS (magic angle spinning) probes spinning were used for ^1^H solid-state NMR. The number of scans used for the ^1^H-NMR spectra was chosen such that the S/N is greater than or equal to 20.

For the liquid samples the procedure was as follow. Once the synthesis was finished an aliquot of the colloidal dispersion in DMF was taken, centrifugated and redispersed in hexane three times to remove residual DMF. The procedure was repeated with MeOD to remove the hexane almost entirety.

For the photocatalytic evaluation: ^1^H-NMR spectra were recorded on a 500 MHz Bruker instrument. Chemical shifts (δ) are reported in parts per million (ppm) relative to TMS (δ (^1^H) = 0.0 ppm). Each spectrum was calibrated using the residual solvent peak as an internal reference, with the ^1^H δ 8.03. The following abbreviations indicate signal multiplicities: s, singlet; d, doublet; m, multiplet. All coupling constants are given in Hz.

**Thermogravimetric analyses (TGA)** were performed using a stream of air in a TA Instruments model TA-STD-Q600 analyzer. The temperature range applied during the analysis spans from 40 °C to 800 °C, with a controlled heating rate of 10°C min^-1^.

**Dynamic Light Scattering (DLS)**. The samples were measured with a Zetasizer Malvern instrument, Multi-Purpose Titrator. According to their Brownian motion, they scatter light in different directions and intensities, thus being able to record their average size by relating the diffusion coefficient to the hydrodynamic diameter using the Stokes-Einstein equation (Equation S1). With it, the average hydrodynamic particle size and the measurement of the size distribution of particle size can be measured.

$d_{H}=\frac{KT}{3\pi\eta D}$	Equation S1

d_H_ = Hydrodynamic diameter	𝜂 = Viscosity

K = Boltzmann constant	D = Diffusion coefficient

T = Temperature

**Field emission scanning electron microscopy (FESEM).** The study was performed on an FESEM JEOL JSM-7900F equipment provided with an energy-dispersive X-ray spectroscopy detector (X-EDS) Ultim Max 170 Oxford Instruments model. The images have been processed by means of Aztec of Oxford instruments software. The sample was prepared by dispersing the material in ethanol and dropped onto silicon wafers and dried.

**Transmission electron microscopy (TEM)** was performed on a TEM JEOL JEM 1400 instrument working at 120 kV or a JEOL JEM 1010 TEM operating at 100 kV. The sample was prepared by dispersing the material in ethanol or DMF (10 μL, 0.5 mg/mL) and dropped onto a formvar/carbon film supported on a 300-mesh copper grid and dried.

**Absorption spectroscopy.** Absorption spectra were recorded with a Perkin Elmer Lambda 1050 UV-Vis-NIR spectrophotometer using quartz cuvettes (1 cm optical path). The absorption spectra of all the nanohybrids in DMF (0.4 mg/mL) were registered from 250 to 800 nm using a step size of 1 nm.

**Steady-state emission spectroscopy.** Photoluminescence spectra were registered using 1 cm light-path quartz cuvettes on an FLS1000 spectrophotometer (Edinburgh Instruments). For UC measurements, a 980 nm CW laser (2W, PSU-III-LED, CNI Optoelectronics Technology Co. Ltd.) was used to irradiate 0.4 mg/mL dispersions in DMF. For recording the CMPBDP emission, samples were excited with a Xe arc lamp (150 W). A PMT-980 photomultiplier (Edinburgh Instruments) was used to detect emissions in the visible (excitation and emission bandwidth of 1 nm). For recording emissions in the NIR window, a PMT 1700 H10330C-75 photomultiplier (Hamamatsu Photonics, K.K.) was used (excitation and emission bandwidth of 10 nm). For measurements in a nitrogen atmosphere, the cuvette containing the dispersions was sealed and bubbled for 10 minutes with a nitrogen flow.

**Time-resolved (TR) emission spectroscopy.** Photoluminescence kinetic profiles were also recorded in quartz cuvettes on the FLS1000 photoluminescence spectrometer (Edinburgh Instruments). The following combinations were used:

· Kinetic profiles at 580 nm: a nanosecond supercontinuous pulsed laser (SuperK Extreme EXU-6, NKT Photonics) at a frequency of 5.5 MHz was used to excite the samples at 500 nm (excitation bandwidth: 5 nm). The signals were registered using a visible photomultiplier (MCP-900, Edinburgh Instruments; emission bandwidth: 5 nm).

· UC kinetic profiles were recorded by exciting the samples with a 980 nm continuous wave laser, controlled by a pulsed electronic laser controller (pulse width: 100 μs; PM2 box, Edinburgh Instruments). A visible photomultiplier (PMT-980, Edinburgh Instruments) collected the signals with an excitation bandwidth of 0.5 nm. Measurements were conducted on 0.4 mg/mL dispersions in DMF.

To ensure a nitrogen atmosphere, the solution in the sealed cuvette was purged with nitrogen gas for at least 10 minutes. Kinetic curve fitting was performed using Fluoracle software.

**Upconversion quantum yield (UCQY).** Absolute quantum yield measurements were performed in sealed quartz cuvettes (1 cm optical path length) using a Quantaurus QY Plus (C13534-11, Hamamatsu Photonics K.K.) equipped with a NIR photoluminescence measurement module (C13684-01, Hamamatsu Photonics K.K.). A 980 nm continuous-wave laser (2.5 W, MDL-III-980, CNI Optoelectronics Technology Co. Ltd.) was used as excitation source. The laser power was adjusted with a variable neutral density disk. The excitation irradiance was calculated based on the laser spot size provided by the manufacturer and the measured laser power using a power meter (PD300-3W, Ophir Optronics Solutions Ltd.) within the integration sphere.

**Solar simulator**. A LCSS500A simple solar simulator (Zolix Instruments Co., Ltd.) was used to replicate the sunlight. The system provides a facula irradiance of 1000 W/m^2^ with a spot size of 25 mm in diameter. This system uses a high-performance XQ500 Xe lamp with a colour temperature of 6000 K to provide a broad spectrum range from 0.25 to 1.8 μm. The steady current was adjusted to 21 A by using the HSP-500XA Steady Current Power Supply external control with a light output ripple < 5% and an AM 1.5 filter.

# 2. Synthesis of Monomer

## 2.1. Synthesis of 4,4-difluoro-2,6-diiodo-1,3,5,7-tetramethyl-4-bora-3a,4a-diaza-s-indacene (I-BODIPY).

4,4-difluoro-1,3,5,7-tetramethyl-4-bora-3a,4a-diaza-s-indacene (411 mg, 1.57 mmol) and iodine (989 mg, 3.90 mmol) were dissolved with 50 mL of EtOH. To the reaction mixture a dissolution of iodic acid (546 mg, 3.18 mmol) dissolved in the minimum amount of water was added dropwise. The reaction was stirred at room temperature for 2 hours. The organic layer was washed with a saturated aqueous Na_2_S_2_O_3_.5H_2_O solution and DCM, dried over MgSO_4_, filtered and the solvents were removed under reduced pressure. Finally, the crude material was purified by flash chromatography using heptane: ethyl acetate (4:1) as eluent. Yielding 629 mg (78%) as red solid. ^[1]^

C_12_H_15_N_2_BF_2_: ^1^H-NMR (400 MHz, CDCl_3_): $\delta$ 2.63 (s, 3H), 2.61 (s, 6H), 2.47 (s, 6H) ppm.

# 3. Synthesis of tetrafluoroborate-stabilized upconverting nanoparticles.

## 3.1. Synthesis of oleate-capped core NaYF_4_: Yb^3+^, Er^3+^ NPs (UC_C_@OA)

UC_C_@OA NPs were synthesized by thermal decomposition with oleic acid (OA) and 1-octadecene (ODE) following slightly modified version of a previously reported protocol.^[2]^ A solution (30 mL) containing 20 mmol of lanthanides (15.6 mmol YCl_3_·6H_2_O, 4 mmol YbCl_3_·6H_2_O, and 0.4 mmol ErCl_3_·6H_2_O) in MeOH was added to a mixture of 300 mL of 1-octadecene and 160 mL of oleic acid in a round-bottom flask under N_2_ atmosphere. The reaction mixture was heated to 160°C before degassing under vacuum for 30 minutes. Then, after cooling down to 60°C, 100 mL of a methanolic solution containing NaOH (50 mmol) and NH_4_F (80 mmol) was added at once. After evaporating the methanol by heating the mixture to 125°C, the temperature was quickly raised to 305°C and maintained for 1.5 hours. The mixture was then cooled to room temperature, and the UCNPs were precipitated by addition of ethanol and recovered by centrifugation at 10000 g for 10 minutes. This process was repeated three times with a mixture of chloroform: ethanol (1:3) and then three times with a mixture of cyclohexane: acetone (1:3). Finally, the precipitate was redispersed in 60 mL of cyclohexane and centrifuged at 1000 g for 3 minutes, retaining the supernatant to discard any large UCNP agglomerates.

## 3.2. S**ynthesis** of oleate-capped core/shell NaYF_4_: Yb^3+^, Er^3+^@NaYF_4_ NPs (UC_CS_@OA).^[3]^

A mixture of 100 mL of 1-octadecene and oleic acid (1:1 ratio) was combined with 15 mL of a methanolic solution containing 10 mmol of YCl₃·6H₂O under nitrogen atmosphere. The mixture was continuously stirred and gradually heated to 150 °C to form a clear and homogeneous solution. Following the evaporation of methanol under vacuum for 30 minutes, the reaction temperature was reduced to 60 °C. At this point, 30 mL of a methanolic solution containing NaOH (25 mmol) and NH₄F (40.5 mmol) was added, still under a nitrogen atmosphere. The reaction mixture was then heated to 130 °C and stirred to ensure methanol and water evaporation. Finally, the temperature was raised to 325°C for 25 minutes. After cooling to room temperature, ethanol was added to allow the precipitation of the UC_CS_@OA, which were recovered by centrifugation at 7000 rpm for 5 min. The white precipitate was washed with three cycles of redispersion followed by centrifugation (7000 rpm, 5 min) in chloroform:ethanol (1:3).

## 3.3. Synthesis of UC_C_ or UC_CS_.

A well-known protocol for the synthesis of tetrabluoroforate-stabilized UCNPs was carried out.^[4]^ Briefly, 10 mL of UC_c_@OA or UC_CS_@OA (25 mg/mL) in cyclohexane, 10 mL of DMF, and 250 mg of nitrosonium tetrafluoroborate (NOBF_4_) were added and stirred vigorously for 60 min. The BF_4_^-^capped UCNPs were recovered from the cyclohexane phase by adding an excess of chloroform followed by centrifugation (7000 g, 7 min). The transparent pellet was redispersed in 5 mL of DMF and precipitated again by adding an excess of chloroform, followed by centrifugation (10000 g, 15 min). Finally, the UC_C_ or UC_CS_ solid was redispersed in 5 mL of DMF and centrifuged at 1000 g for 1 minutes to remove larger aggregates.

# 4. Synthesis of UC@BBS

In a round-bottom flask, the corresponding UC_C_ or UC_CS_ (100 mg, synthesized as described in 3.3 section) were dispersed in DMF. 4-bromobenzenesulfonic acid monohydrated (200 mg) was added and, the reaction volume was adjusted to 20 mL with DMF. The reaction mixture was stirred at room temperature for 48 hours. Then, the BBS-functionalized UCNPs were isolated by centrifugation (13000 g, 30 min). The resulting white precipitate was washed once with a DMF:chloroform (1:5 v/v) followed by a second centrifugation step under identical conditions. The obtained UC_C_@BBS or UC_CS_@BBS nanohybrids were subsequently redispersed in 3 mL of DMF.

# 5. Synthesis of UC@CMPBDP

To prepare UC@CMPBDP with different polymer loadings (5, 10 and 15 wt.%), the monomers, 1,3,5-triethynylbenzene (5.5x10^-3^ mmol, 2.75x10^-3^ mmol or 1.4x10^-3^ mmol, respectively) and diiodo-BODIPY (5x10^-3^ mmol, 2.5x10^-3^ mmol or 1.25x10^-3^ mmol, respectively), were dissolved in DMF (5.5 mL) and triethylamine (5.5 mL) and degassed by argon bubbling for 10 minutes. [Pd(PPh_3_)_4_] (0.2x10^-3^, 0.1x10^-3^ or 0.05x10^-3^mmol, respectively) and CuI (0.4x10^-3^, 0.2x10^-3^ or 0.1x10^-3^mmol, respectively) were added to the organic phase. The organic phase was added dropwise to a Pyrex tube containing 2.25 mL of UC_C_@BBS or UC_CS_@BBS in DMF (33.3 mg/mL). The resulting mixture was degassed for 20 minutes, then heated at 80 ℃ for 4 days under gentle stirring. After completion of the reaction, the mixture was subjected to a mild centrifugation/washing cycle (1x 1500 rpm), followed by three additional centrifugation/washing cycles at higher speed (3x 9000 rpm) in DMF for washing. The DMF supernatants from each washing cycle were discarded. The resulting solid was redispersed in DMF (7 mL). Then, 10 mg of NaCN were added to the **UC@CMPBDP-X** dispersions in DMF and stirred for 10 h. Finally, the dispersions were subjected to three further centrifugation/washing cycles (3x 9000 rpm, in DMF) to lead to the final pure DMF dispersions of **UC@CMPBDP-X** ready to be used in the characterization and photocatalysis experiments. Additionally, aliquots of **UC@CMPBDP-X** dispersions were filtered, washed and dried to perform their structural characterization in the solid state.

# 6. Nanohybrids Characterization

Figure S1. TEM images of A) NaYF_4_: Yb^3+^ (20%), Er^3+^ (2%), UC_C_, and B) NaYF_4_: Yb^3+^ (20%), Er^3+^ (2%)@NaYF_4_, UC_CS_**.** Scale bars: 100 nm. C) and D) Size distribution histograms of UC_C_ and UC_CS_, respectively.

Figure S2. Absorption spectra of BBS (black line), UC_C_@BBS (red line), and UC_CS_@BBS (blue line) in DMF.

Figure S3. TEM images of UC_C_@BBS (A) and UC_CS_@BBS (B). Scale bars: 100 nm.

Figure S4. FTIR-ATR spectra of **UC_CS_@CMPBDP-X** (X = 5 and 15).

Figure S5. Thermogravimetric analyses under air atmosphere of (A) **UC_CS_@BBS,** (B) **UC_CS_@CMPBDP-5**, (C) **UC_CS_@CMPBDP-10**, (D) **UC_CS_@CMPBDP-15**, (E) **UC_C_@BBS** and (F) **UC_C_@ CMPBDP-15.**

Figure S6. Powder XRD spectrum of **UC_CS_@CMPBDP-10.**

Figure S7.HR-SEM images of **UC_CS_@CMPBDP-5** (A), **UC_CS_@CMPBDP-10** (B) and **UC_CS_@CMPBDP-15** (C) at different magnifications.

Figure S8. UC_CS_@CMPBDP-X (X= 5 (A and B); X =10 (C and D); X= 15 (E and F)) images taken by FESEM (left side) as well as FESEM images taken by back scattered electrons detection mode (right side).

Figure S9. TEM images of (A) UC_C_@CMPBDP-15, (B) UC_CS_@CMPBDP-5, (C) UC_CS_@CMPBDP-10, (D) UC_CS_@CMPBDP-15 and (E-G) TEM images of UC_CS_@CMPBDP-10 at different magnifications.

## Table S1. Solvodynamic sizes of the UCNP nanohybrids calculated from 5 independent DLS measurements.

| Hybrid | DLS | |
| --- | --- | --- |
|  | Size (nm) | Population (%) |
| UC_CS_@BBS | 47±10 | 100 |
| UC_CS_@CMPBDP-5 | 82±14 | 100 |
| UC_CS_@CMPBDP-10 | 86±16 | 100 |
| UC_CS_@CMPBDP-15 | 95±18 | 100 |

Figure S10. Solvodynamic particle size distribution of dispersions UC_Cs_@BBS (cyan), UC_CS_@CMPBDP-5 (pink), UC_CS_@ CMPBDP-10 (green), and UC_C_@CMPBDP-15 (brown), in DMF at a concentration of 0.04 mg/mL at room temperature.

**Figure S11.** Nitrogen sorption isotherms (filled circles: adsorption; open circles: desorption) of UC_CS_@CMPBDP-10 at 77 K.

Figure S12. (A) Absorption and (B) emission (λ_exc_= 350 nm) spectra of a CMPBDP dispersion in DMF.

Figure S13. A) Emission (λ_exc_= 980 nm) of CMPBDP (orange line), UC_C_@BBS (dotted line), UC_CS_@BBS (dashed line) in DMF and B) Ratio of the integrated areas under the emission spectra between 642-682 nm (I_642-682_) and 514-564 nm (I_514-564_) for the different samples (λ_exc_= 980 nm). Calculated from UC emission spectra after secondary inner-filter effect correction.

Figure S14. Upconversion quantum yield (λ_exc_= 980 nm) in DMF calculated in the emission range between 400 and 700 nm of UC_CS_@BBS (purple circle), UC_C_@BBS (purple square), UC_CS_@ CMPBDP-11 (red circle), and UC_C_@CMPBDP-15 (green square).

Figure S15- Kinetic profiles of the nanohybrids in DMF under different atmospheric conditions: (A) air and (B) nitrogen (N₂), monitored at 540 nm upon excitation at 980 nm. Color code: UC_C_@BBS (purple), UC_C_@CMPBDP-15 (green), UC_CS_@BBS (pink), UC_CS_@CMPBDP-5 (black), UC_CS_@CMPBDP-10 (red), and UC_CS_@CMPBDP-15 (blue).

Table S2. Lifetimes of the nanohybrids and pristine UCNPs obtained from the kinetic profiles at λ_exc_=980 nm and λ_em_=540 nm in air. <τ_av_> estimated as the amplitude-weighted average lifetime.

|  | τ_1_ /μs | τ_2_ /μs | <τ_av_> /μs | χ^2^ |
| --- | --- | --- | --- | --- |
| UC_C_@BBS | 95.5 ± 0.7 | - | - | 1.0864 |
| UC_C_@CMPBDP-15 | 82.8 ± 0.4 | - | - | 1.1094 |
| UC_CS_@BBS | 255.1 ± 1.8 (89.1%) | 797.5 ± 37.2 (10.9%) | 275 ± 3 | 0.8691 |
| UC_CS_@CMPBDP-5 | 220.6 ± 0.9 (94.4%) | 867 ± 20 (5.6%) | 230 ± 2 | 1.2710 |
| UC_CS_@CMPBDP-10 | 241.9 ± 0.9 (87.4%) | 681 ± 11 (12.6%) | 263.4 ± 1.4 | 1.0632 |
| UC_CS_@CMPBDP-15 | 213.4 ± 0.6 (92.0%) | 743 ± 17 (8.0%) | 226.3 ± 0.9 | 1.1693 |

Table S3. Lifetimes of the nanohybrids obtained from the kinetic profiles at λ_exc_=980 nm and λ_em_=540 nm in N_2_.

|  | τ_1_ /μs | τ_2_ /μs | <τ_av_> /μs | χ^2^ |
| --- | --- | --- | --- | --- |
| UC_C_@CMPBDP-15 | 81.6 ± 0.3 | - | - | 1.0807 |
| UC_CS_@CMPBDP-5 | 241 ± 2 (99.9 %) | 632 ± 30 (0.1%) | 241 ± 2 | 0.9999 |
| UC_CS_@CMPBDP-10 | 239.9 ± 5.8 (83.4%) | 493 ± 63 (16.6%) | 262 ± 11 | 1.0284 |
| UC_CS_@CMPBDP-15 | 212.7 ± 0.7 (91.6%) | 717.4 ± 44 (8.4%) | 226 ± 2 | 1.3022 |

Figure S16. Kinetic profiles (λ_exc_= 980 nm) at 580 nm of the nanohybrids in DMF: UC_CS_@CMPBDP-5 (black), UC_CS_@CMPBDP-10 (red), UC_CS_@CMPBDP-15 (blue), and UC_C_@CMPBDP-15 (green).

Table S4. Lifetimes of the nanohybrids obtained from the kinetic profiles at λ_exc_=980 nm and λ_em_=580 nm in N_2_.

|  | τ_1_ /μs | χ^2^ |
| --- | --- | --- |
| UC_C_@CMPBDP-15 | 92.5 ± 1.2 | 0.9411 |
| UC_CS_@CMPBDP-5 | 257 ± 5 | 0.9410 |
| UC_CS_@CMPBDP-10 | 240 ± 5 | 0.4491 |
| UC_CS_@CMPBDP-15 | 185 ± 3 | 0.7477 |

Figure S17. Kinetic profiles of the nanohybrids in DMF under different atmospheric conditions: (A) air and (B) nitrogen (N₂), monitored at 660 nm upon excitation at 980 nm. Color code: UC_C_@BBS (purple), UC_C_@CMPBDP-15 (green), UC_CS_@BBS (pink), UC_CS_@CMPBDP-5 (black), UC_CS_@CMPBDP-10 (red), and UC_CS_@CMPBDP-15 (blue).

Table S5. Lifetimes of the nanohybrids and pristine UCNPs obtained from the kinetic profiles at λ_exc_=980 nm and λ_em_=660 nm in air.

|  | τ_1_ /μs | χ^2^ |
| --- | --- | --- |
| UC_C_@BBS | 259.1 ± 0.6 | 0.9975 |
| UC_C_@CMPBDP-15 | 246.2 ± 0.5 | 0.9997 |
| UC_CS_@BBS | 382.5 ± 0.5 | 0.9999 |
| UC_CS_@CMPBDP-5 | 363.7 ± 0.8 | 0.9978 |
| UC_CS_@CMPBDP-10 | 337.2 ± 0.7 | 0.9972 |
| UC_CS_@CMPBDP-15 | 366.5 ± 0.6 | 1.0010 |

Table S6. Lifetimes of the nanohybrids obtained from the kinetic profiles at λ_exc_=980 nm and λ_em_=660 nm in N_2_.

|  | τ_1_ /μs | χ^2^ |
| --- | --- | --- |
| UC_C_@CMPBDP-15 | 251.3 ± 0.3 | 0.9999 |
| UC_CS_@CMPBDP-5 | 363.9 ± 0.5 | 0.9998 |
| UC_CS_@CMPBDP-10 | 345.1 ± 0.9 | 0.9963 |
| UC_CS_@CMPBDP-15 | 364.4 ± 0.5 | 0.9973 |

Figure S18. A) NIR emission (λ_exc_= 980 nm) of 0.4 mg/mL dispersions in DMF. Colour code: CMPBDP (grey), UC_CS_@BBS (purple), UC_CS_@CMPBDP-5 (black), UC_CS_@CMPBDP-10 (red), UC_CS_@CMPBDP-15 (blue), UCC@BBS (pink), and UC_C_@CMPBDP-15 (green). B) Integrated areas under the emission spectra between 1450-1620 nm (I_1450-1620_) for the different samples (λ_exc_= 980 nm).

Table S7. Lifetimes of the nanohybrids obtained from the kinetic profiles at λ_exc_=980 nm and λ_em_=1550 nm in air.

|  | τ_1_ / ms | χ^2^ |
| --- | --- | --- |
| UC_C_@CMPBDP-15 | 2.518 ± 0.003 | 5.1844 |
| UC_CS_@CMPBDP-5 | 4.617 ± 0.010 | 1.1769 |
| UC_CS_@CMPBDP-10 | 5.029 ± 0.002 | 2.5490 |
| UC_CS_@CMPBDP-15 | 4.620 ± 0.005 | 2.3004 |

Table S8. Lifetimes of the nanohybrids obtained from the kinetic profiles at λ_exc_=980 nm and λ_em_=1550 nm in N_2_.

|  | τ_1_ / ms | χ^2^ |
| --- | --- | --- |
| UC_C_@CMPBDP-15 | 2.570 ± 0.003 | 7.6525 |
| UC_CS_@CMPBDP-5 | 5.027 ± 0.008 | 0.9992 |
| UC_CS_@CMPBDP-10 | 4.614 ± 0.019 | 0.9999 |
| UC_CS_@CMPBDP-15 | 4.252 ± 0.016 | 1.9993 |

# 7. Inner-filter effect

Inner-filter effect (IFE) is an optical interference phenomenon that can significantly influence fluorescence measurements, often leading to inaccurate intensity readings.^[5]^ IFE is categorized in two types:

i) Type I IFE, which is caused mainly by the absorption of the excitation light by substances, usually non-fluorescent, present in the solution. That limits the amount of light reaching the desired fluorophore
ii) Type II IFE, which is a consequence of the re-absorption of emitted light by other components of the solution, decreasing the final intensity emission.

IFE correction is essential to ensure reliable quantification and interpretation of fluorescence data.^[6]^ In our described system, TYPE II IFE was observed as CPP absorbs in the green up-converted emission region of UCNPs. To correct the UC emission spectra and account for this re-absorption, the following equation has been applied:^[5]^

$$I_{corr}(\lambda)=I_{exp}(\lambda)\cdot{10}^{A(\lambda)}$$

Where I_corr_(λ) and I_exp_(λ) are the corrected and experimental emission spectra profile at any wavelength, respectively, and A (λ) is the absorption spectrum of the sample at any wavelength.

# 8. Photocatalytic reaction

The as-synthesized UC@CMPBDP-X nanohybrids were evaluated as photocatalyst in the oxidation of α-terpinene following the concentration, solvent and irradiation time optimized for other UCNHs used as photocatalysts.^[7]^ Briefly, 200 μL of an oxygen-saturated solution of α-terpinene (0.06 M) and the corresponding UC@CMPBDP nanohybrid in deuterated DMF (DMF-d₇) were irradiated with a 980 nm continuous-wave laser diode (5W, 308 W/cm^2^) for 8 hours under continuous stirring and temperature control at 20°C (Scheme 2). The reaction mixture was centrifuged (10000 g, 10 min) to remove the nanohybrids. Then, the supernatant was analyzed by ^1^H-NMR using CHCl_3_ as internal standard. The photocatalytic efficiency of the nanohybrids upon NIR excitation was evaluated by calculating the yield of formation of oxidation photoproducts, such as ascaridole and several aldehydes and carboxylic acids as well as the competing autooxidation or dehydrogenation pathway that leads to *p*-cymene and isomerization pathway that leads to *γ*− and *β*-terpinene (see section 11).^[8]^ The reaction conditions, conversion and products yields are shown in Table 1.

# 9. Recyclability of the photocatalysts

The precipitated nanohybrid, recovered by centrifugation of the crude reaction mixture, was redispersed with 200 μL of a freshly prepared α-terpinene solution (0.06 M) in DMF. The mixture was bubbled with oxygen for 10 minutes and subsequently irradiated for 8 h with a 980 nm laser diode. The reaction products were analyzed by ^1^H-NMR as previously described.^[7]^

# 10. Solar simulator

A DMF-d7 solution containing α-terpinene (0.06 M) and the selected catalyst, without UC_CS_@CMPBDP-10 (35 mg/mL) or CPP (3.5 mg/mL), was irradiated in a double-walled Pyrex glass reactor. The irradiation source was an LCSS500A Simple Solar Simulator (Zolix Instruments Co., Ltd.), which produced a collimated beam with a uniform light spot.

The reactor was sealed and kept under an oxygen-saturated atmosphere. A water-circulating cooling system maintained the temperature at 20°C. The samples were irradiated under these conditions for 8 hours.

# 11. Calculation of reaction yields

The supernatant obtained from centrifugation of the crude reaction mixture was directly diluted in 430 μL of DMF-*d_7_*, and subsequently analysed by ^1^H-NMR spectroscopy. The product yields were estimated by comparing the integral of the olefinic protons of *α*-terpinene (5.61 ppm, 2H, m, 2×C*H*=); *γ*-terpinene (5.8 ppm, 2H, m, 2×C*H*=); *β*−terpinene (5.4 ppm, 2H, m, C*H*_2_=; 6.97 ppm, 1H, m, C*H*=); ascaridole (6.56 ppm, 1H, d, *J* = 8.6 Hz, C*H*=; 6.45 ppm, 1H, d, *J* = 8.6 Hz, C*H*=), aromatic protons of *p*-cymene (7.13 ppm, dd, *J* = 8.0 Hz, J = 1 Hz, 4H, CAr-H); the aldehyde signals of 3*-Isopropyl-6-oxohept-2-enal* (**4**) or 3,5 di*methyl-6-oxooct-2-enal* (**5**) (10.06 ppm, d, 1H, *J* = 7.7 Hz, C*H*O) and their carboxylic acid derivatives (11.5 ppm, d, 1H, *J* = 7.7 Hz) (see Scheme S1).

**Scheme S1**. Photooxidation and other thermal pathways of α-terpinene upon irradiation of UC@CMPBDP nanohybrids (λ_exc_= 980 nm) under oxygen. Note that the H used to determine the product from ^1^H-NMR are in red.

## 11.1. ^1^H-NMR characterization

*The complete H-NMR characterization of the most abundant reactants and products observed is:*

*α-terpinene*: ^1^H-NMR (500 MHz, DMF-*d_7_*) δ (5.8 ppm, 2H, m, 2×C*H*=), 2.37-2.20 (m, 1H, -C*H*(CH_3_)_2_), 2.07 (m, 4H, 2×CH_2_), 1.74 (s, 3H), 1.00 (d, *J* = 6.8 Hz, 6H, -CH(C*H*_3_)_2_).

*ascaridole*: ^1^H-NMR (500 MHz, DMF-*d_7_*) δ 6.56 (d, *J* = 8.6 Hz, 1H, C*H*=), 6.45 (d, *J* = 8.6 Hz, 1H, C*H*=), 1.93-1.85 (m, 2H, -CH_2_-), 1.86-1.78 (m, 1H, -C*H*(CH_3_)_2_), 1.57 – 1.44 (m, 2H, -CH_2_-), 1.29 (s, 3H, C*H*_3_-C_Ar_), 0.96 (d, 3H, *J* = 6.9 Hz, -CH(CH_3_)-C*H*_3_), 0.94 (d, 3H, *J* = 6.9 Hz, -CH(CH_3_)-C*H*_3_). NMR data matched with those reported in the literature.^[9]^

*p-cymene*: ^1^H-NMR (500 MHz, DMF-*d_7_*) δ 7.13 (dd, *J* = 8.0 Hz, *J* = 1 Hz, 4H, C_Ar_-*H*), 2.61 (m, 1H, -C*H*(CH_3_)_2_), 2.28 (s, 3H, C*H*_3_-C_Ar_), 1.20 (d, *J* = 6.9 Hz, 6H, -CH(C*H*_3_)_2_). NMR data matched with those reported in the literature.^[10]^

## 11.3. Recyclability assays

**Table S9.** Estimated reaction yields of the photooxidation of the α-terpinene using 980 nm excitation and recycling UC_CS_@CMPBDP-5 nanohybrids as photocatalysts

| Entry | PC | cycle | Light source | [PC]  (mg/mL) | Overall Conversion (%) | Photoproduct Conversion (%) | Yield (%) | | | |
| --- | --- | --- | --- | --- | --- | --- | --- | --- | --- | --- |
|  |  |  |  |  |  |  | Ascaridole | *p*-cymene | γ-terpinene | Aldehydes & carboxylic |
| 1 | UC_CS_@CMPBDP-5 | 1^st^ | NIR | 35 | 59.0 | 42 | 39.0 | 9.3 | 19.5 | 32.2 |
| 2 |  | 2^nd^ |  |  | 57 | 40 | 36.8 | 10.2 | 18.9 | 33.3 |
| 3 |  | 3^rd^ |  |  | 66 | 44 | 33.3 | 10.9 | 21.2 | 33.3 |
| 4 |  | 4^th^ |  |  | 40 | 22 | 35.5 | 20.8 | 22.5 | 20 |
| 5 |  | 5^th^ |  |  | 42 | 22.8 | 34 | 21.9 | 21.9 | 20.2 |

**Table S10.** Estimated reaction yields of the photooxidation of the α-terpinene using 980 nm excitation and recycling UC_CS_@CMPBDP-10 nanohybrids as photocatalysts

| Entry | PC | cycle | Light source | [PC]  (mg/mL) | Overall Conversion (%) | Photoproduct Conversion (%) | Yield (%) | | | |
| --- | --- | --- | --- | --- | --- | --- | --- | --- | --- | --- |
|  |  |  |  |  |  |  | Ascaridole | *p*-cymene | *γ*-terpinene | Aldehydes & carboxylic |
| 1 | UC_CS_@CMPBDP-10 | 1^st^ | NIR | 35 | 58.9 | 26.9 | 31.6 | 27.2 | 27.2 | 14.1 |
| 2 |  | 2^nd^ |  |  | 65.0 | 36.0 | 29.2 | 20.0 | 24.6 | 26.2 |
| 3 |  | 3^rd^ |  |  | 36.0 | 26.2 | 61.1 | 7.2 | 17.5 | 11.7 |
| 4 |  | 4^th^ |  |  | 46.2 | 34.9 | 65.8 | 6.5 | 18.0 | 9.7 |
| 5 |  | 5^th^ |  |  | 45.3 | 35.5 | 61.8 | 6.0 | 15.0 | 16.6 |

# 12. References

[1]	T. Yogo, Y. Urano, Y. Ishitsuka, F. Maniwa, T. Nagano, “Highly Efficient and Photostable Photosensitizer Based on BODIPY Chromophore” *J. Am. Chem. Soc.* **2005**, *127*, 12162–12163.

[2]	Z. Li, Y. Zhang, “An efficient and user-friendly method for the synthesis of hexagonal-phase NaYF_4_:Yb, Er/Tm nanocrystals with controllable shape and upconversion fluorescence” *Nanotechnology* **2008**, *19*, 345606.

[3]	N. Estebanez, J. Ferrera-González, L. Francés-Soriano, R. Arenal, M. González-Béjar, J. Pérez-Prieto, “Breaking the Nd^3+^-sensitized upconversion nanoparticles myth about the need of onion-layered structures” *Nanoscale* **2018**, *10*, 12297–12301.

[4]	A. Dong, X. Ye, J. Chen, Y. Kang, T. Gordon, J. M. Kikkawa, C. B. Murray, “A Generalized Ligand-Exchange Strategy Enabling Sequential Surface Functionalization of Colloidal Nanocrystals” *J. Am. Chem. Soc.* **2010**, *133*, 998–1006.

[5]	L. Ceresa, J. Kimball, J. Chavez, E. Kitchner, Z. Nurekeyev, H. Doan, J. Borejdo, I. Gryczynski, Z. Gryczynski, “On the origin and correction for inner filter effects in fluorescence. Part II: secondary inner filter effect -the proper use of front-face configuration for highly absorbing and scattering samples” *Methods Appl. Fluoresc.* **2021**, *9*, 35005.

[6]	M. Kubista, R. Sjöback, S. Eriksson, B. Albinsson, “Experimental correction for the inner-filter effect in fluorescence spectra” *Analyst* **1994**, *119*, 417–419.

[7]	L. Francés-Soriano, D. Bellezza, J. Ferrera-González, M. González-Béjar, J. Pérez-Prieto, “NIR-triggered photooxygenation of α-terpinene with upconversion nanohybrids” *Nanoscale Adv.* **2024**, *6*, 5889–5896.

[8]	F. Ronzani, N. Costarramone, S. Blanc, A. K. Benabbou, M. Le Bechec, T. Pigot, M. Oelgemöller, S. Lacombe, “Visible-light photosensitized oxidation of α-terpinene using novel silica-supported sensitizers: Photooxygenation vs. photodehydrogenation” *J. Catal.* **2013**, *303*, 164–174.

[9]	D. Prat, A. Wells, J. Hayler, H. Sneddon, C. R. McElroy, S. Abou-Shehada, P. J. Dunn, “CHEM21 selection guide of classical- and less classical-solvents” *Green Chem.* **2016**, *18*, 288–296.

[10]	F. Lévesque, P. H. Seeberger, “Highly Efficient Continuous Flow Reactions Using Singlet Oxygen as a ‘Green’ Reagent” *Org. Lett.* **2011**, *13*, 5008–5011.
